# A novel co-culture model for investigation of the effects of LPS-induced macrophage-derived cytokines on brain endothelial cells

**DOI:** 10.1371/journal.pone.0288497

**Published:** 2023-07-13

**Authors:** Junling Yang, Yinchuan Li, Ambuj Bhalla, Mark Maienschein-Cline, Ken-ichiro Fukuchi

**Affiliations:** 1 Department of Cancer Biology and Pharmacology, University of Illinois College of Medicine, Peoria, Illinois, United states of America; 2 Institute of Reproductive Medicine, Medical School of Nantong University, Nantong, Jiangsu, People’s Republic of China; 3 Research Informatics Core, Research Resources Center, University of Illinois Chicago, Chicago, Illinois, United States of America; Eötvös Loránd Research Network Biological Research Centre, HUNGARY

## Abstract

In order to study effects of macrophage-derived inflammatory mediators associated with systemic inflammation on brain endothelial cells, we have established a co-culture system consisting of bEnd.3 cells and LPS-activated Raw 264.7 cells and performed its cytokine profiling. The cytokine profile of the co-culture model was compared to that of mice treated with intraperitoneal LPS injection. We found that, among cytokines profiled, eight cytokines/chemokines were similarly upregulated in both in vivo mouse and in vitro co-culture model. In contrast to the co-culture model, the cytokine profile of a common mono-culture system consisting of only LPS-activated bEnd.3 cells had little similarity to that of the in vivo mouse model. These results indicate that the co-culture of bEnd.3 cells with LPS-activated Raw 264.7 cells is a better model than the common mono-culture of LPS-activated bEnd.3 cells to investigate the molecular mechanism in endothelial cells, by which systemic inflammation induces neuroinflammation. Moreover, fibrinogen adherence both to bEnd.3 cells in the co-culture and to brain blood vessels in a LPS-treated animal model of Alzheimer’s disease increased. To the best of our knowledge, this is the first to utilize bEnd.3 cells co-cultured with LPS-activated Raw 264.7 cells as an in vitro model to investigate the consequence of macrophage-derived inflammatory mediators on brain endothelial cells.

## Introduction

Increasing studies on clinical subjects and experimental animal models suggest that systemic inflammation promotes neurodegeneration and cognitive deterioration in neurological diseases such as Alzheimer’s disease (AD) [1–3]. Aging is the biggest among a number of risk factors for AD and characterized by chronic/systemic low-grade inflammation, called “inflammaging” [4–6]. Diabetes, hyperlipidemia, hypertension, and obesity are modifiable risk factors of AD and depicted by a chronic/systemic low-grade inflammation [7–10]. In addition to modifiable risk factors, large-scale genome-wide association studies (GWAS) on late-onset AD subjects have identified a dozen genetic risk variants that are involved in immune/inflammation responses, highlighting the importance of inflammatory responses in the pathogenesis of late-onset AD [11, 12]. Indeed, Lopez-Rodriguez *et al*. demonstrated that acute inflammatory events induced by systemic LPS produce exaggerated chemokine and IL-6 pathway responses in astrocytes in an AD animal model, compared with the wild type control cohort. Moreover, microglia with enhanced expression of IL-1β are induced by systemic LPS administration in the hippocampal dentate gyrus in an AD animal model but not in the wild type control cohort [13]. The findings infer that astrocytes/microglia are primed by amyloid deposition in AD brain and that second challenges such as LPS administration and infections amplify inflammatory responses. Lonnemann *et al*. [14] reported that transgenic expression of human anti-inflammatory IL-37 limits neural abnormalities, neuroinflammation, and behavioral deficits in LPS-treated wild-type mice as well as in AD model mice. However, the efficacy of anti-inflammatory drugs in preventing and treating dementia including AD is still uncertain [15, 16].

The disruption of the blood-brain barrier (BBB) by systemic inflammation and by brain infiltration of immune cells/inflammatory mediators increases neuroinflammation and exacerbates clinical symptoms and neuronal death in patients with neurodegenerative diseases [17–20]. Because the BBB contains brain vascular endothelial cells as a main element [21] and because peripheral administration of LPS causes systemic inflammation [22–25], LPS-treated endothelial cells are widely used to investigate the mechanisms underlying the BBB dysfunction associated with systemic inflammation [26–28]. Activation of endothelial cells is triggered by high levels of LPS via a receptor complex of TLR4, CD14 and MD2, resulting in endothelial cell apoptosis and potentially severe vascular collapse in sepsis [26]. Inversely, LPS directly induces endothelial cells to express cytoprotective proteins, A1 and A20 via CD14-dependent pathways requiring activation of NF-κB [29]. In addition to TLRs, a diversity of receptors are expressed on the endothelial cells, which include receptors for IL-1β, IL-6, and tumor necrosis factor-α (TNFα) [30–32]. Intensive profiling of cytokines and cell-surface immune receptors reveals that the responses of human cerebral microvascular endothelial cells (hCMVEC) to IL-1β significantly differ from those to TNFα [33]. Therefore, it is difficult to investigate the complex molecular events associated with systemic inflammation, which take place at the BBB, by individually examining the effects of inflammatory mediators on brain endothelial cells.

In order to better understand the effects of macrophage-derived inflammatory mediators associated with systemic inflammation on brain endothelial cells, we have established a co-culture system consisting of bEnd.3 cells and LPS-activated Raw 264.7 cells in this study. Furthermore, we have determined the effects of LPS-induced acute peripheral inflammation on brain amyloid load and BBB permeability in an AD mouse model and utilized the co-culture system to investigate a potential mechanism underlying the phenotypic changes in vivo.

## Material and methods

### Experimental animals

A congenic C57BL/6J line of AD model mice, B6.Cg-Tg (APPswe, PSEN1dE9) 85Dbo/J mice was purchased from Jackson Laboratory (Jackson Laboratory, Bar Harbor, ME) and propagated by crossing transgenic males with C57BL/6 females. The genotyping for the APPswe/PS1dE9 transgenes was performed by the PCR-based method provided by the Jackson Lab. B6.Cg-Tg mice are defined as “APP mice”. In this study, 14-month-old APP male mice (n = 6/group) were administrated with intraperitoneal injection of 0.5 mg/kg LPS once (Cat#: L2880, Sigma-Aldrich, St. Louis, MO) or PBS served as controls. The mice were housed in group cages with bedding and maintained on a 12/12 hr light-dark cycle (on at 6 AM) under a specific pathogen free condition with access to irradiated food of the 5LG4 (LabDiet) and water ad libitum. All animal protocols used for this study were prospectively reviewed and approved by the Institutional Animal Care and Use Committee of the University of Illinois at College of Medicine at Peoria.

### Murine CSF collection

CSF was isolated from the cisterna magna compartment using the method previously described [34]. Mice were anesthetized by ketamine and xylazine and fixed face down on a narrow platform. An incision was made from the top of the skull to the dorsal thorax. The musculature from the base of the skull to the first vertebrae was carefully removed to expose the meninges overlying the cisterna magna. The surrounding area was gently cleaned with PBS using cotton swabs to remove any residual blood or other interstitial fluid. The arachnoid membrane covering the cistern was punctured with a 29 gauge insulin syringe. A polypropylene narrow bore pipette was immediately placed in the hole to collect CSF. As the primary CSF exiting the compartment was collected, a second collection was performed after the cistern was refilled within 2 min. About 10 to 15 μl CSF was collected from each mouse. Then mice were transcardially perfused with cold PBS and their brains were quickly removed.

### Immunohistochemical and immunofluorescent analyses

After CSF collection followed by cardiac perfusion with cold PBS, the neocortices and hippocampi of the left hemispheres were separately dissected and stored at -80°C for further studies. The right hemispheres were fixed in 4% paraformaldehyde for 48 h, stored overnight in 30% sucrose in 0.1 M PBS and frozen in Tissue-Tek optimal cutting temperature compound. For detection of fibrillar Aβ plaque, tissue sections were stained in 1% thioflavin S (Sigma-Aldrich, St. Louis, MO) and rinsed with 70% ethanol. After washing with distilled water, the sections were mounted in ShandonTM Immu-MountTM. Histomorphometry was performed for quantification of amyloid deposits using a Zeiss Axioscan and Image Pro 10 image analysis software (Media Cybernetics, Silver Spring, MD) capable of color segmentation and automation via programmable macros for immunohistochemistry staining. Three coronal brain sections from each mouse were analyzed, each separated by an approximately 400-μm interval, starting at 1.3 mm posterior to the bregma to caudal. Both neocortex and hippocampus were found in all the brain sections and analyzed separately. Stained areas were expressed as a percentage of total neocortex or hippocampus, respectively. For immunofluorescent staining, the coronal sections of the brains were immune-stained with anti-CD31 antibody (Cat# 557355, 1: 500 dilution, BD Pharmingen™, Franklin Lakes, NJ) and anti-fibrinogen (Cat# A0080, 1:300 dilution, Dako, Japan). Sections in 1ml of antigen retrieval buffer (citrate buffer, PH 6.0) (Abcam, Cambridge, MA) in a 2ml tube were performed antigen retrieval at 85°C in a water bath and cool down at room temperature for 2h. After washing with 0.1 M Tris buffer and 0.1M TBS, sections were blocked with 1% BSA in 0.1 M TBS with 0.5% triton-X-100 (TBS-T) and then incubated with primary antibodies in 1% BSA in TBS-T for 48 h at 4°C. For the negative controls, slides were processed without primary antibody. After rinsing, the sections were incubated with donkey-anti-rat antibody (Cell Signaling Technology, Danvers) and chicken-anti-rabbit antibody (Invitrogen, Carlsbad, CA) for 2 h at room temperature. Cell nuclei were counterstained with 4′, 6-Diamidino-2-phenylindole dihydrochloride (DAPI). The sections were mounted in ShandonTM Immu-MountTM (Thermo Fisher Scientific, Waltham, MA). Histomorphometry was performed for quantification of the ratio of fibrinogen positive area to CD31 positive area using Olympus FV3000 confocal microscope (Olympus American Inc, Waltham, MA) and Image J. Data were expressed as mean ± standard error of the mean (SEM).

### Quantification of brain and CSF Aβ by ELISA

The left neocortex and hippocampus were removed from storage at −80°C, lysed using the Bio-Plex cell lysis kit (Bio-Rad Laboratories, Hercules, CA, USA) and homogenized in Bullet Blender® (Thermo Fisher Scientific, Waltham, MA) according to the manufacturer’s protocol and centrifuged at 12,000×g for 10 min at 4°C. The supernatants containing buffer-soluble Aβ were collected and the protein concentrations in the supernatants were determined by Bio-Rad Protein Assay (Bio-Rad Laboratories, Hercules, CA, USA). The pellets containing buffer-insoluble Aβ were further homogenized in guanidine hydrochloride (final concentration, 5 M) in Bullet Blender and then rock shaken for 3–4 h at room temperature. Levels of buffer-soluble and insoluble Aβ in the left neocortex and hippocampus and Aβ in the CSF were quantified by Aβ42 and Aβ40 enzyme-linked immunosorbent assay (ELISA) kits (Invitrogen, Carlsbad, CA, USA) according to the manufacturer’s protocol.

### Cell culture

**Fig 1:**
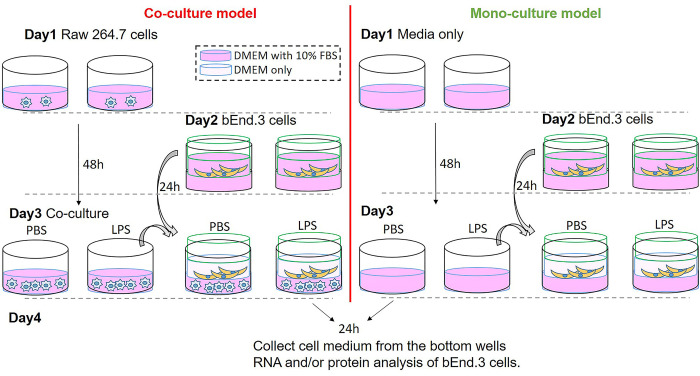
Schematic diagram of Raw 264.7 and bEnd.3 cell co-culture and mono-culture. For co-culture model, Raw 264.7 cells were plated on a 6-well plate on day1, and bEnd.3 cells were added to the inside compartment of Transwell inserts sitting in another 6-well plate on day2. On day3, Raw 264.7 cells were treated with PBS or LPS (100 ng/ml) followed by adding the Transwell inserts with bEnd.3 cells. After 24 h co-culture, cell media and bEnd.3 cells were harvest for further analysis. The mono-culture was done side by side with the co-culture.

The RAW 264.7 (ATCC® TIB-71™) cells and bEnd.3 cells (ATCC® CRL2299™) were purchased from ATCC and cultured in ATCC-formulated Dulbecco’s modified eagle’s medium (DMEM) containing 10% fetal bovine serum (FBS) (ATCC) in a humidified 37°C incubator with a 5% CO_2_ atmosphere. The Raw 264.7 and bEnd.3 cells co-culture model is outlined in Fig 1. On day 1, RAW 264.7 cells were seeded in 6-well plates (3 x 10^5^cells/well) in 10% FBS DMEM. On day 2, bEnd.3 cells were seeded in Corning™ Transwell™ Permeable Polycarbonate Membrane Inserts (pore size: 3μm) (Thermo Fisher Scientific, Waltham, MA) (2 x 10^5^ cells/insert in 10% FBS DMEM. On day 3, RAW 264.7 cells were treated with PBS or 100ng/ml LPS in 10% FBS DMEM. Meanwhile, bEnd.3 cells in the transwell inserts were replaced with fresh DMEM and co-culture with Raw 264.7 cells treated with PBS or LPS. As for mono-culture system, the conditions were same except no Raw 264.7 seeded in the wells (Fig 1). Culture media were collected for proteome profiling at 24 h after LPS or PBS treatment. The bEnd.3 cells were analyzed at 24 h after LPS or PBS treatment.

### Cytokine profiling assay

Cell culture media were harvested 24h after LPS treatment from the bottom wells. One ml of each cell culture medium supernatant was collected by centrifugation at 500 x g for 5 minutes to remove particulates. Cytokines in the culture supernatant were analyzed by Mouse Cytokine Array Panel A kit (Cat# ARY006, R&D Systems, Inc. Minneapolis, MN) by following the instructions provided by the manufacturer. This array contains 40 different capture antibodies printed in duplicate to simultaneously detect differences in relative levels of cytokines and chemokines between samples. Positive signals detected by chemiluminescent reagents in the kit were exposed to the X-ray film. The pixel densities of the signals were determined by densitometric scanning using an HP Scanjet G3010 Photo Scanner and Image J V1.40 (NIH, MD). The average pixel density of the pair of duplicate spots representing each cytokine expression levels was determined by subtracting an average background signal (negative control spots as a background value) from each spot.

### RNA sequencing and bioinformatics analysis

Total RNA was isolated from bEnd.3 cells using the Direct-zol^TM^ RNA kit (Zymo Research Corporation, Irvine, CA). Sequencing on DNBSEQ high-throughput platform was performed at BGI Tech Solution NGS lab (Hong Kong, CHN). Raw reads were aligned to reference genome mm10 using STAR [35]. ENSEMBL genes were quantified using FeatureCounts [36] as raw read counts. Differential expression statistics (fold-change and p-value) were computed using edgeR [37, 38] using generalized linear models to compute differential expression due to genotype and treatment in a two-factor design, including an interaction term. Pairwise comparisons between groups were also computed using the “exactTest” function. Gene expression was normalized using TMM factors in edgeR. In all cases p-values were adjusted for multiple testing using the false discovery rate (FDR) correction of Benjamini and Hochberg [39]. PCA plots were generated from log-scaled normalized expression. We identified gene expression patterns with dependent effects of genotype and treatment through a clustering analysis. Differentially expressed genes (DEGs) for clustering were selected based on interaction term FDR<0.2. Differential profiles of these genes were computed as the product of the log fold-change and -log p-value for each pairwise comparison, and normalized by dividing by their standard deviation. We then performed k-means clustering on these profiles with ten random initializations on a range of cluster numbers k (2 to 20). We determined the optimal k by evaluating clustering reproducibility statistics for each set of results, similar to the approach outlined by Senbabaoglu and co-workers [40]: for each k, we computed the pair-wise distance between clustering results as the fraction of co-clustered feature pairs across two clustering results relative to the number of co-clustered feature pairs within each result individually. This difference was averaged across all result pairs for each value of k, and the largest k with an average distance less than 1e-5 was selected as the ideal clustering granularity–k = 4 was determined to be the best number of clusters. Functional interpretation and ontology enrichment analysis of DEGs lists were performed using online tool Enrichr (Enrichr, https://maayanlab.cloud/Enrichr/) database [41].

### Validation of genes expression in brain and bEnd.3 cells

Total RNA from the left hemispheres and the bEnd.3 cells was isolated using the Direct-zol^TM^ RNA kit (Zymo Research Corporation, Irvine, CA). Total RNA (500ng) from each sample was reverse transcribed using iScript™ Reverse Transcription Supermix kit (Bio-Rad Laboratories, Hercules, CA, USA) to synthesize cDNA. Primer pairs used for real-time PCR are shown in Table 1. The data were analyzed using the comparative Ct method (2^-ΔΔCT^) with GAPDH as the normalizer.

**Table 1:** Primer DNA sequences.

Gene	Forward primer (5’ to 3’)	Reverse primer (5’ to 3’)
**CCL2**	TTAAAAACCTGGATCGGAACCAA	GCATTAGCTTCAGATTTACGGGT
**CCL3**	CCTCTGTCACCTGCTCAACA	GATGAATTGGCGTGGAATCT
**CCL4**	CCCACTTCCTGCTGTTTCTC	GAGGAGGCCTCTCCTGAAGT
**CCL5**	ATATGGCTCGGACACCACTC	GGGAAGCTATACAGGGTCA
**CD36**	GGAGCCATCTTTGAGCCTTCA	GAACCAAACTGAGGAATGGATCT
**CSF1**	GCTGGATGATCCTGTTTGCTA	GACACAGGCCTTGTTCTGCT
**CXCL1**	ATAATGCCCTTTTACATTCTTTAA	AGTCCTTTGAACGTCTCTGTCC
**CXCL10**	GCTGCCGTCATTTTCTGC	TCTCACTGGCCCGTCATC
**CX3CL1**	CCTCACTAAAAATGGTGGCAAG	ATGTCAGCCGCCTCAAAAC
**ICAM1**	CCTCTGCTCCTGGCCCTGGT	CGGACTGCTGTCCTCCCCGA
**IL-1α**	AGGAGAGCCGGGTGACAGTA	AACTCAGCCGTCTCTTCTTCAGA
**IL-1β**	GCAACTGTTCCTGAACTCAACT	ATCTTTTGGGGTCCGTCAACT
**IL-6**	TAGTCCTTCCTACCCCAATTTCC	TTGGTCCTTAGCCACTCCTTC
**IRF7**	GAGACTGGCTATTGGGGGAG	GACCGAAATGCTTCCAGGG
**TNFα**	CCCTCACACTCAGATCATCTTCT	GCTACGACGTGGGCTACAG
**BACE1**	GGAACCCATCTCGGCATCC	TCCGATTCCTCGTCGGTCTC
**PS1**	GGTGGCTGTTTTATGTCCCAA	CAACCACACCATTGTTGAGGA
**IDE**	AATCCGGCCATCCAGAGAATA	GGGTCTGACAGTGAACCTATGT
**ACE**	AGGTTGGGCTACTCCAGGAC	GGTGAGTTGTTGTCTGGCTTC
**NEP**	CTCTCTGTGCTTGTCTTGCTC	GACGTTGCGTTTCAACCAGC

### Western blot

The bEnd.3 cell lysates were prepared by using radioimmune precipitation assay (RIPA) lysis buffer containing complete mini-protease-inhibitor and phosphatase inhibitor cocktail tablets (Roche Diagnostics Corporation, Indianapolis, IN) and centrifuged at 12,000 x g for 10 min at 4°C for collecting supernatants. After determining protein concentrations, the proteins were electrophoresed under reducing conditions in 10% SDS–PAGE gels and transferred to PVDF membranes. The membranes were incubated overnight at 4°C with anti-Claudin-5 (Cat# 35–2500, 1:1000 dilution, Thermo Fisher Scientific, Waltham, MA), anti-STAT1 (Cat#14994S, 1:1000 dilution, Cell Signaling Technology, Danvers), anti-STAT2 (Cat#72604S, 1:1000 dilution, Cell Signaling Technology, Danvers), anti-IFITM3 (Cat#ab15592, 1:1000 dilution, Abcam, Cambridge, MA), and anti-GAPDH antibody (Cat#MAB374, 1:2000 dilution, Sigma-Aldrich, St. Louis, MO). Specific bands were visualized by an enhanced chemiluminescence system (Amersham, Arlington Heights, IL). The optical densities of the protein bands were determined by densitometric scanning using an HP Scanjet G3010 Photo Scanner and Image J V1.40 (NIH, MD). GAPDH was used to normalize expression levels of genes of interesting.

### Statistical analysis

Data were expressed as mean ± SEM. Analysis of variance (ANOVA) and two-tailed Student’s t-test were used to determine the intergroup significant difference. P < 0.05 was considered statistically significant.

## Results

### Comparison of cytokine expression profiling between endothelial/macrophage co-culture and endothelial mono-culture stimulated by LPS

**Fig 2:**
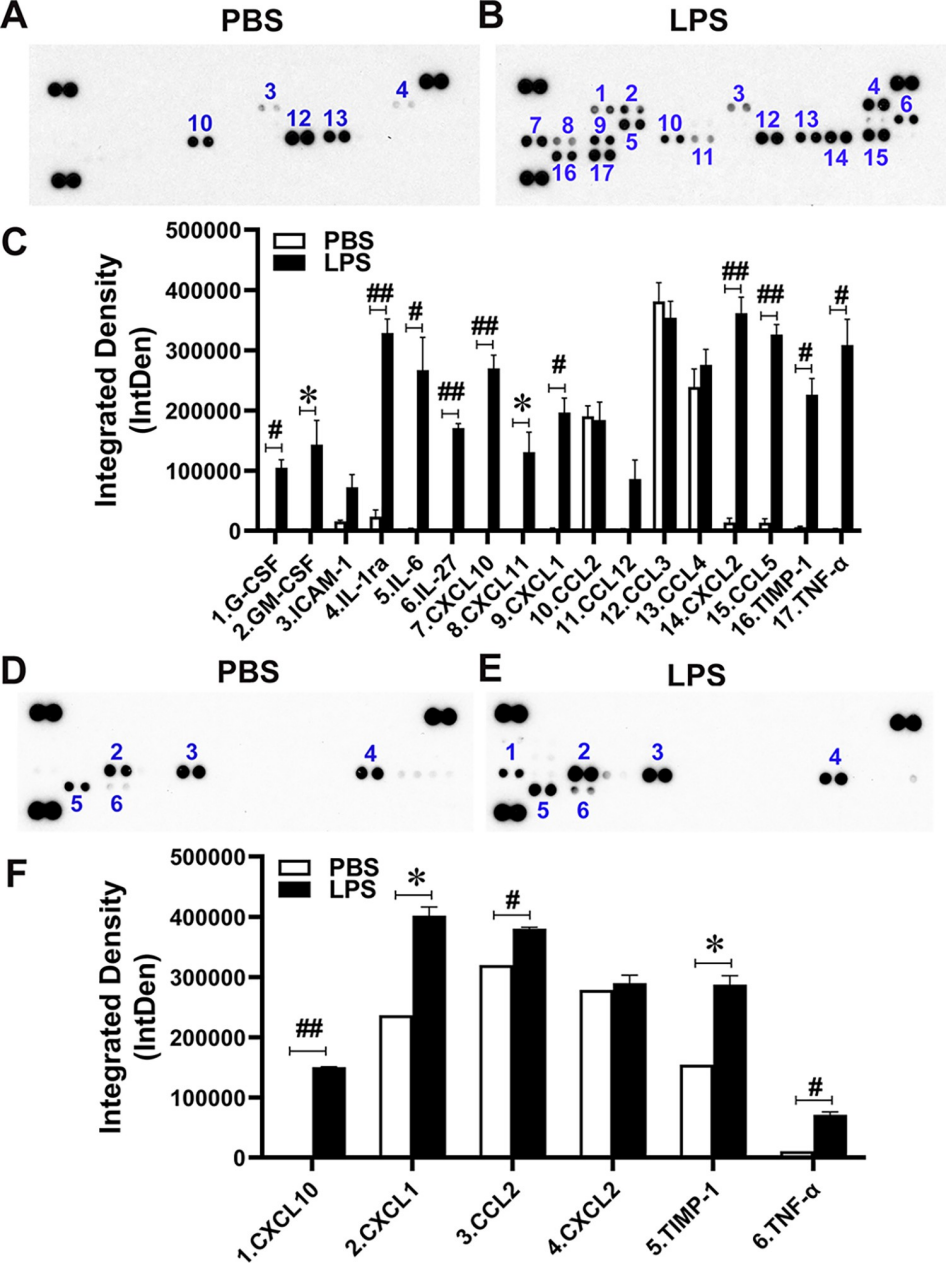
LPS induces inflammatory cytokine secretion in vitro. Analysis of chemokines/cytokines in media of bEnd.3 and Raw 264.7 co-culture (A, B, and C) and media of bEnd.3 mono-culture (D, E, and F) by proteome profiler mouse cytokine array. C and F are semi quantitative analysis of chemokines/cytokines. The identification numbers labeled right above or below the signal dots in Fig 2A and 2B correspond to the numbers assigned to cytokines and chemokines in Fig 2C (co-culture model), and in Fig 2D and 2E correspond to those in Fig 2F (mono-culture). (Data from three independent experiments, *P < 0.05, #P < 0.01, and ##P < 0.001).

Because proinflammatory cytokines/chemokines induced by systemic inflammation alter the BBB integrity and functions [42–44] and because brain endothelial cells, one of the components of the BBB, are first responders to peripheral inflammatory molecules including LPS, bEnd.3 cells were co-cultured with LPS-activated Raw 264.7 cells to mimic in vivo models of LPS-induced systemic inflammation (Fig 1). On day 4, 1ml of cell medium from the bottom wells where Raw 264.7 cells were located was applied to the cytokines profiling analysis. In the co-culture model, twelve cytokines were significantly upregulated by LPS, which include G-CSF (P = 0.002), GM-CSF (P = 0.025), IL-1ra (P < 0.001), IL-6 (P = 0.008), IL-27 (P < 0.001), CXCL10 (P < 0.001), CXCL11 (P = 0.018), CXCL1 (P = 0.001), CXCL2 (P < 0.001), CCL5 (P < 0.001), TIMP-1 (P = 0.001), and TNFα (P = 0.002) (Fig 2A–2C). Among these upregulated molecules, 8 cytokines/chemokines increased more than 40-fold in the co-culture model system. In contrast, a mono-culture of bEnd.3 cells stimulated with LPS showed such large increases in only one cytokine, CXCL10 (P < 0.001) (Fig 2D–2F), suggesting that most cytokines upregulated in the co-culture model were secreted from LPS-stimulated Raw 264.7 cells. It is noteworthy that many cytokines are increased in systemic inflammation induced by intraperitoneal injection of LPS [45]. Therefore, we performed the profiling of plasma cytokines in mice subjected to intraperitoneal injection of LPS. First, we determined the time-course changes of plasma TNFα and IL-6 levels after LPS intraperitoneal injection. We found that the levels of plasma TNFα were 1307.7 ± 255.5 pg/ml at 2h and undetectable at 24h after LPS injection. The levels of plasma IL-6 were 41427.7 ± 5658.4 pg/ml at 2h and 629.0 ± 334.4 pg/ml at 24h after LPS injection (S1A Fig). Both cytokines were undetected by ELISA in mice treated with PBS. Accordingly, we performed the cytokines profiling assay using plasma harvested at 2 h after intraperitoneal administration of LPS or PBS. As in the case with cytokine profiling in the co-culture model (Fig 2A–2C), 12 cytokines significantly increased by LPS, including G-CSF (P < 0.001), IL-1ra (P < 0.001), IL-6 (P < 0.001), CXCL10 (P < 0.001), CXCL1 (P < 0.001), M-CSF (P = 0.008), CCL2 (P < 0.001), CCL3 (P < 0.001), CCL4 (P < 0.001), CXCL2 (P < 0.001), TIMP-1 (P < 0.001), and TNFα (P < 0.001) (S1B–S1D Fig), suggesting that LPS-induced macrophage-derived cytokines may have more profound effects on brain endothelia cells than LPS itself.

### Upregulation of cytokine signaling in the co-culture model of systemic inflammation by differential expression analysis

**Fig 3:**
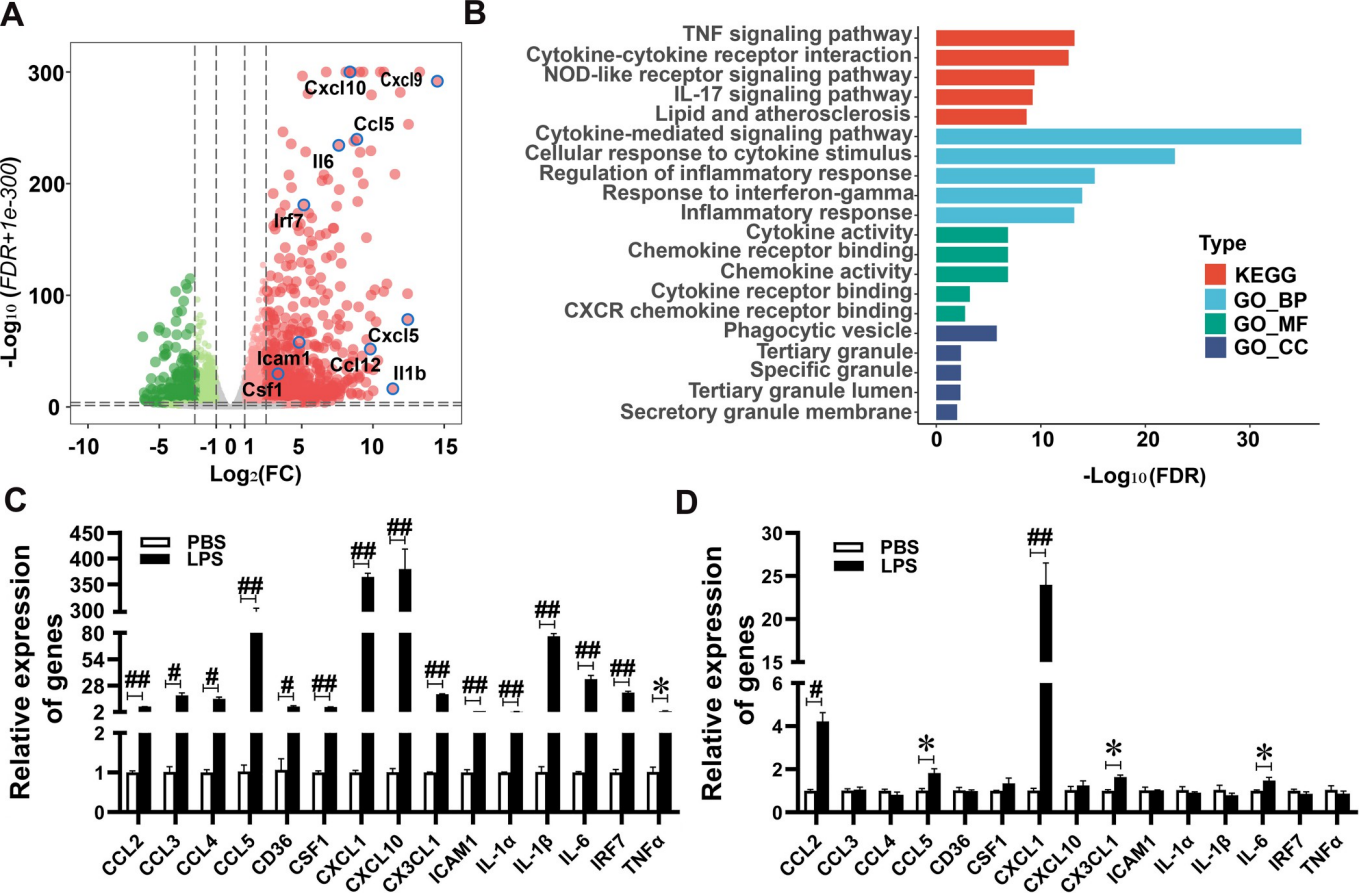
LPS-teated Raw 264.7 cells trigger a distinct transcriptomic response in bEnd.3 cells in vitro co-culture model. (A), Volcano plot showing upregulated genes under the filtering option, fold change (FC) > 2, and FDR < 0.05. (B), Cluster profiler functional annotation showing KEGG and GO enrichment analysis of DEGs groups reflects main categories of terms, GO_BP, gene ontology biological process; GO_MF, gene ontology molecular function; and GO_CC, gene ontology cellular component. Vertical and horizontal axes represent terms and P values of the corresponding terms, respectively. C, Quantitative real-time PCR (qRT-PCR) assays were used to validate genes of interest revealed by DEGs. (D), Quantitative real-time PCR (qRT-PCR) were used to determine the genes in bEnd.3 cells only (mono-culture). (Data from three independent experiments, *P < 0.05, #P < 0.01, and ##P < 0.001).

To further investigate the mechanisms by which cytokines/chemokines secreted from Raw 264.7 cells are involved in potentially pathogenic changes of brain endothelial cells, we performed RNA-seq of bEnd.3 cells after 24 h of co-culture with LPS-activated Raw 264.7 cells, bEnd.3 cells co-cultured with PBS-treated Raw 264.7 cells served as a control. Compared with the control group, a total of 7867 genes were found to be DEGs with the false discovery rate (FDR) < 0.05 cut off, which include 4159 upregulated and 3708 downregulated genes in bEnd.3 cells co-cultured with LPS-activated Raw 264.7 cells as shown in the volcano plot (Fig 3A and S1 Table). When the filtering option with fold change > 2, FDR < 0.05, and LPS and PBS group mean counts > 2 was applied, 2004 genes were upregulated and 1519 genes were downregulated, respectively (S2 Table). The upregulated genes were subjected to a comprehensive gene set enrichment analysis using the Enrichr web server (http://amp.pharm.mssm.edu/Enrichr) (S3 Table). The top five signaling pathways in KEGG and Gene Ontology (GO) enrichment analysis were shown in Fig 3B and S4 Table. The cytokine-mediated signaling pathway (GO: 0019221) was found to be the most significant by the enrichment analysis of the upregulated genes and involved 176 DEGs (S3 Table_GO_BP). We selected 15 genes for real-time PCR validation in bEnd.3 cells from the co-culture as well as mono-culture systems, which showed higher levels of expression and significant differences. In consistent with RNA-seq data, CCL2 (P < 0.001), CCL3 (P = 0.002), CCL4 (P = 0.001), CCL5 (P < 0.001), CD36 (P = 0.003), CSF1 (P < 0.001), CXCL1 (P < 0.001), CXCL10 (P < 0.001), CX3XL1 (P < 0.001), ICAM1 (P < 0.001), IL-1α (P < 0.001), IL-1β (P < 0.001), IL-6 (P < 0.001), IRF7 (P < 0.001), and TNFα (P = 0.018) were significantly increased in co-culture systems (Fig 3C) but only five genes, including CCL2 (P = 0.002), CCL5 (P = 0.022), CXCL1 (P < 0.001), CX3CL1 (P = 0.004), and IL-6 (P = 0.041), increased in mono-culture systems (Fig 3D). These results suggest that peripheral inflammation may induce the cytokine/chemokine secretion from bEnd.3 cells through cytokine signaling pathways, which alter gene expression of the endothelial cells.

### Potential signaling pathways involved with alteration of endothelial cells in the co-culture model

**Fig 4:**
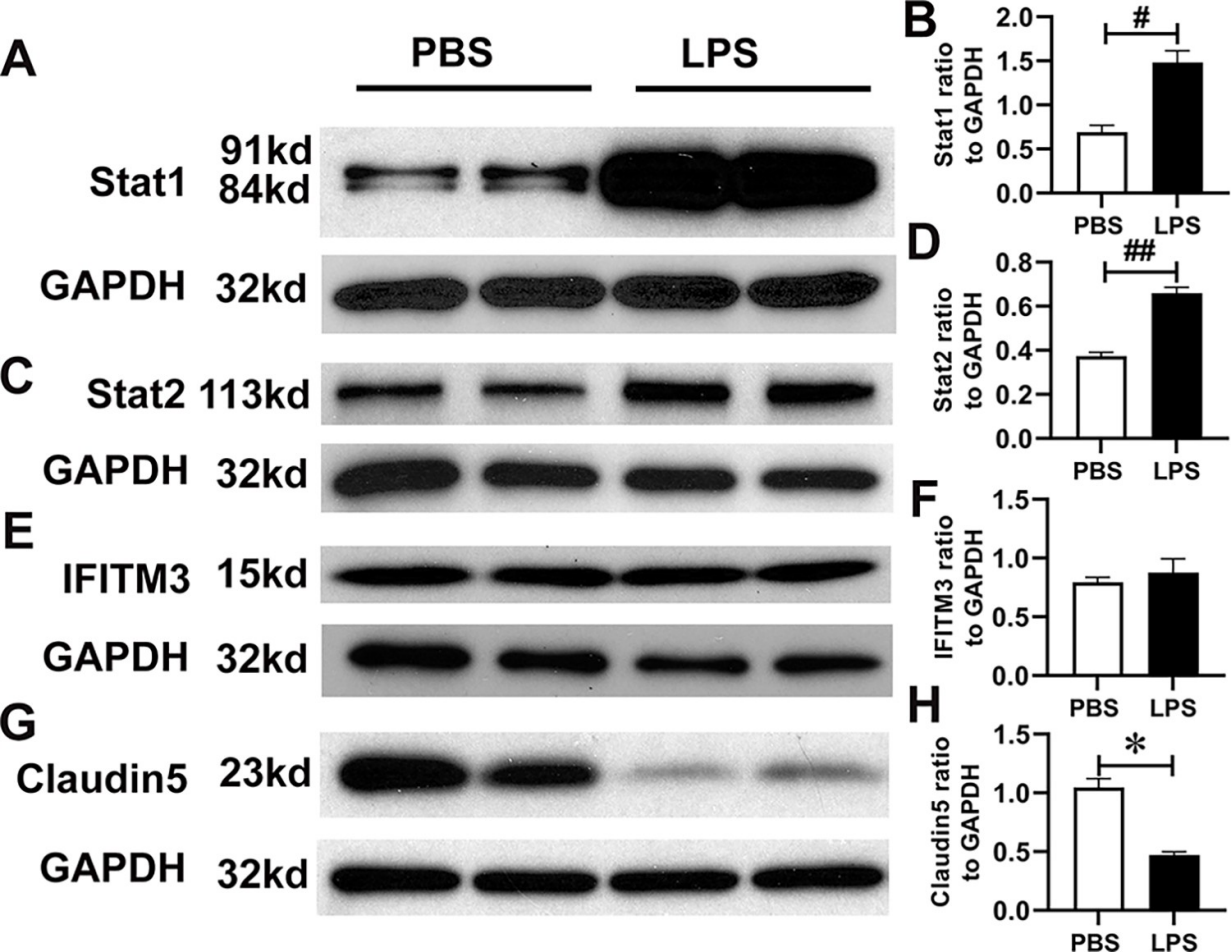
Western blot analysis of cytokine signaling pathways in bEnd.3 cells co-cultured with LPS-induced Raw 264.7 cells. The bEnd.3 cell lysates were analyzed by western blotting using Stat1, Stat2, IFITM3 and Claudin-5 antibodies (A, C, E, and G) and bar graph represents the results of densitometric analysis (B, D, F, and H). Representative blots from three independent experiments are shown. (*P < 0.05, #P < 0.01, and ##P < 0.001).

Although cytokines trigger multiple intracellular signaling pathways, the Janus kinase (JAK)-signal transducer and activator of transcription (STAT) pathway plays critical roles in regulating immune responses [46, 47]. Our RNA-seq data indicated that expression levels of STAT1 and STAT2 were significantly enhanced in the bEnd.3 cells co-cultured with LPS-activated Raw264.7 cells (S2 Table). Thus, we further determined the level of proteins STAT1 and STAT2 by western blot. As expected, the levels of STAT1 and STAT2 were significantly increased in the bEnd.3 cells co-cultured with LPS-induced Raw 264.7 cells, P = 0.002 and P< 0.001, respectively (Fig 4A–4D). Interferon-induced transmembrane protein 3 (IFITM3) is widely expressed in animal tissues, which serves as the first line of defense against virus infection [48–50]. LPS [51], type I and II interferons and IL6 [52] can upregulate IFITM3 expression in cells. Increased expression of IFITM3 has been reported in stroke and other inflammatory conditions in the brain [53], including AD [54]. The RNA-seq analysis revealed increases in IFITM3 expression in bEnd.3 cells co-cultured with LPS-activated Raw264.7 cells but such increases were not found at protein levels by western blot analysis (Fig 4E and 4F). Cytokines/chemokines and other secreted molecules during systemic inflammation may impair the BBB integrity [55]. At the BBB, the most abundant tight junction protein is claudin-5 that is involved in progression of neurodegenerative disorders including AD [56]. We found that claudin-5 was significantly reduced in bEnd.3 cells co-cultured with LPS-induced Raw 264.7 cells (P < 0.001) (Fig 4G and 4H), suggesting that downregulated claudin-5 may cause impairment of the BBB. However, the levels of STAT1, STAT2, claudin-5 were not significantly changed by LPS in mono-culture model (S2A–S2F Fig).

### The effect of peripheral acute inflammation induced by LPS on brain amyloid load

**Fig 5:**
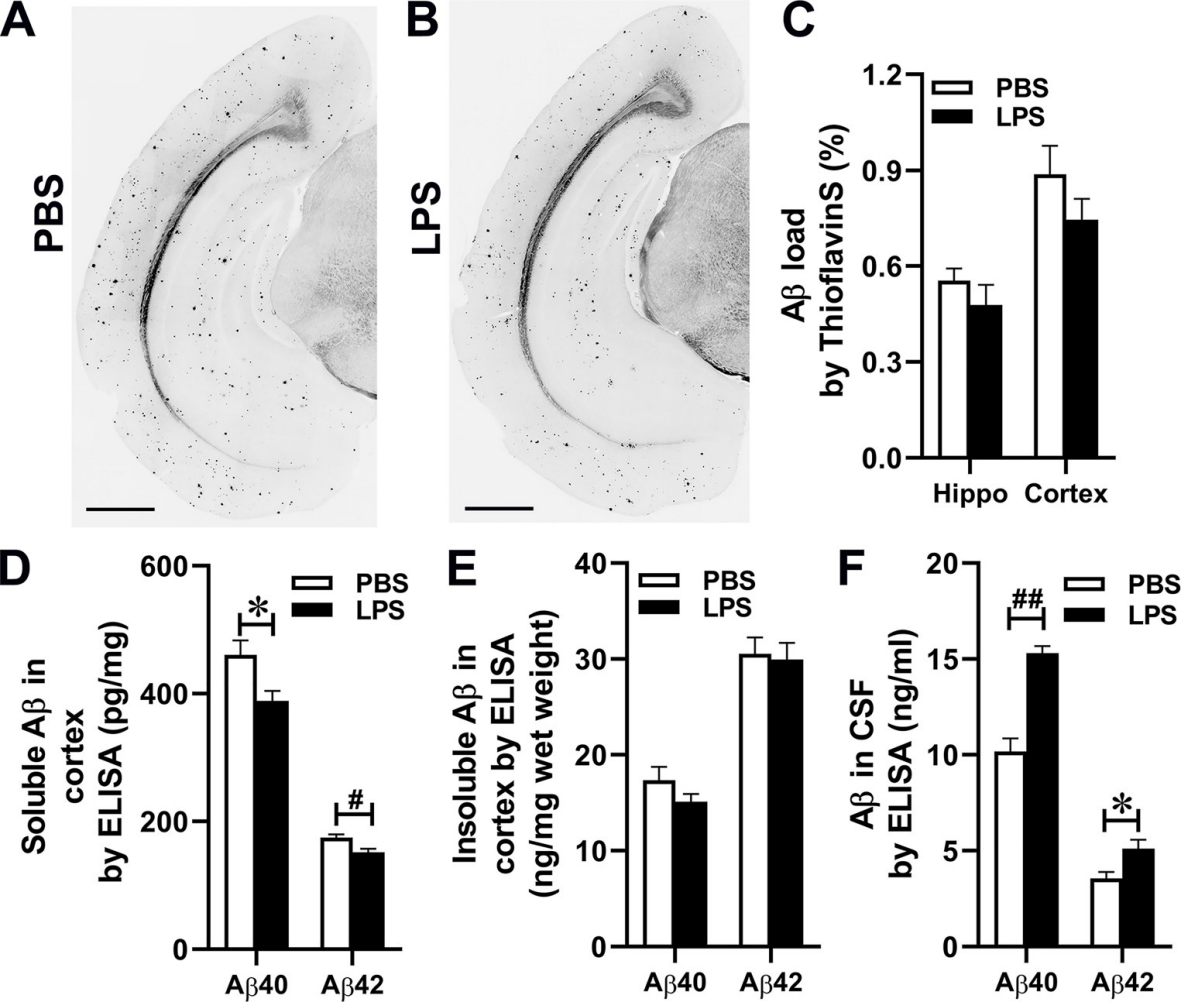
Systemic LPS administration reduces soluble Aβ levels in the brain and increases Aβ levels in CSF. (A & B), Fibrillar Aβ deposits in the brain were visualized by thioflavin S staining. Scale bars: 1mm. (C), Average percentages of areas showing thioflavin S positive staining measured by morphometry in the cortex and hippocampus are shown. (D & E), The levels of soluble and insoluble Aβ40 and Aβ42 in the cortex and hippocampus were measured by ELISA. (F), The levels of Aβ40 and Aβ42 in CSF were measured by ELISA. Data are shown as mean ± SEM. (n = 6 male mice/group, *P < 0.05, #P < 0.01).

Because peripheral inflammation induced by intraperitoneal administration of LPS increases Aβ formation and decreases cognitive function [57] and because the injection numbers, dose, and route of LPS produce variation in pathological reactions [58], we determined the effect of peripheral inflammation on brain Aβ load in APP mice. Fourteen-month-old male APP mice were subjected to intraperitoneal injection of 0.5 mg/Kg LPS or PBS and, 24 h after the administration, euthanized for CSF and brain collection. Aβ deposits in the brain sections were visualized by thioflavin S fluorescence and quantitated by morphometry (Fig 5A–5C). The areas positive for thioflavin S fluorescence showed no statistic differences between the groups in the hippocampus and neocortex (0.48 ± 0.06% and 0.75 ± 0.07% in LPS-injected mice, and 0.55± 0.04% and 0.88 ± 0.09% in PBS injected mice, respectively). Surprisingly, cortical soluble Aβ40 and Aβ42 levels in LPS-injected mice significantly decreased compared with those in control mice (Aβ40, 388.57 ± 15.71 ng/mg protein and Aβ42, 151.58 ± 5.47 ng/mg protein in LPS-injected mice; Aβ40, 460.98 ± 22.29 ng/mg protein, P = 0.020, and Aβ42, 174.80 ± 4.79 ng/mg protein, P = 0.0096, in PBS injected mice) (Fig 5D) while no differences of Aβ40 and Aβ42 levels in insoluble fractions were found between the two groups (Fig 5E). Moreover, both CSF Aβ40 and Aβ42 levels dramatically increased in LPS-injected mice (15.31 ± 0.36 and 5.12 ± 0.47 ng/ml, respectively) compared with those in control mice (10.17 ± 0.69 and 3.55 ± 0.35 ng/ml, respectively) (P < 0.001 and P = 0.023, respectively) (Fig 5F). These results imply that acute peripheral inflammation can alter brain Aβ levels.

### The evaluation of fibrinogen deposition in vitro cell culture model and in the mouse brain

**Fig 6:**
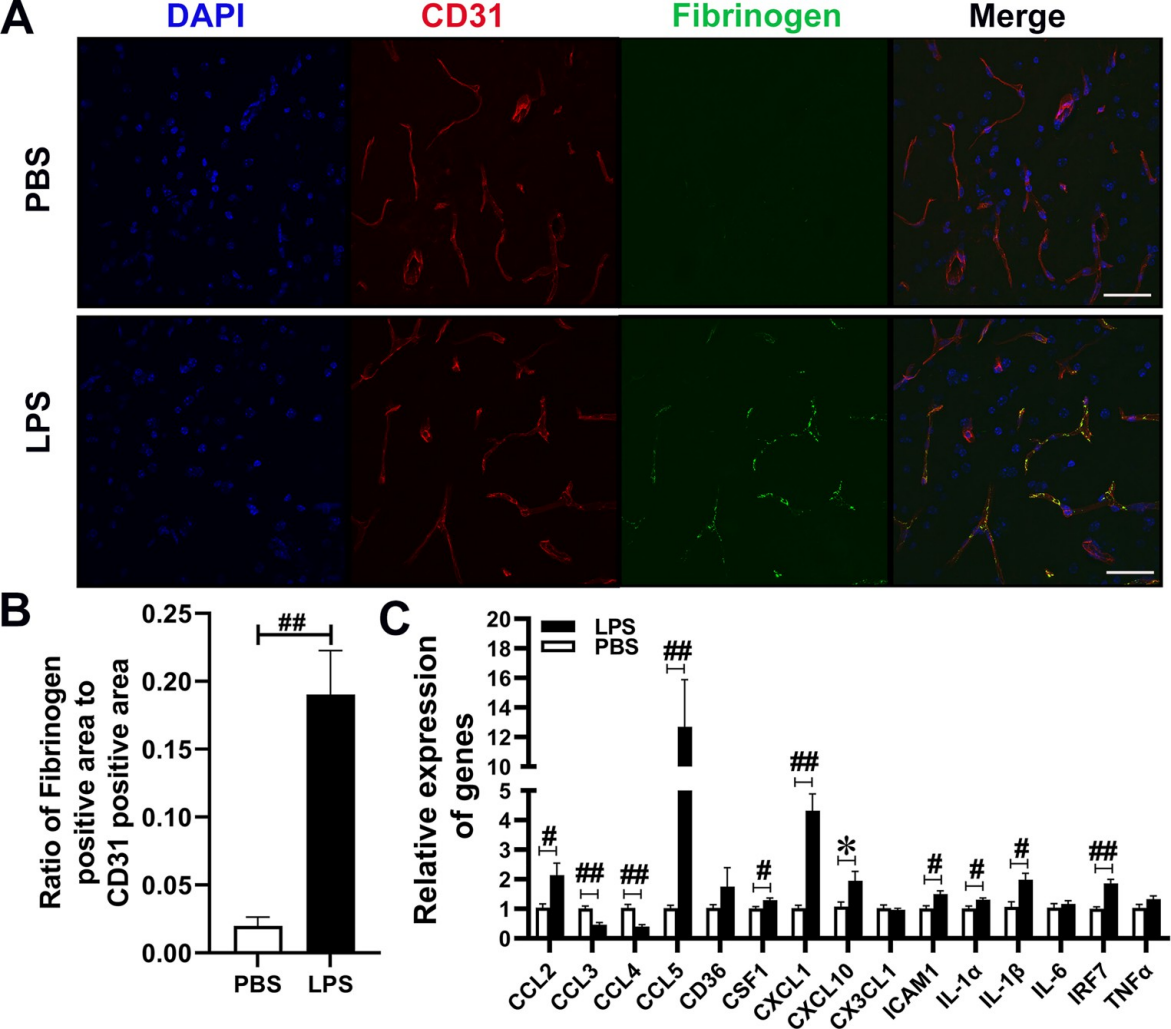
Systemic LPS administration increases fibrinogen adherence to the wall of the brain blood vessels. (A), Immunofluorescent staining of endothelial marker, CD31 (red) and fibrinogen (green). Scale bar: 50μm. (B), The ratios of fibrinogen positive area to CD31 positive area measured by Image J in the hippocampus are shown. (C), Quantitative real-time PCR were used to determine expression levels of the genes of interest in the hippocampus (n = 6 male mice/group, *P < 0.05, #P < 0.01, and ##P < 0.001).

To investigate the potential mechanism underlying the decreases and increases in Aβ levels in the brain and CSF, respectively, we evaluated the integrity of the BBB. Fibrinogen leakage via the impaired BBB induces rapid microglial responses in vivo [59] and fibrinogen deposits in the brain parenchyma has been used as evidence for the BBB breakdown [60]. Accordingly, we performed fibrinogen and CD31 co-immunostaining in our co-culture model as well as in LPS-treated APP mice. In the co-culture model, we found that fibrinogen deposition significantly increased in bEnd.3 cells co-cultured with LPS-treated Raw 264.7 cells (0.04 ± 0.02 vs 0.29 ± 0.03, P<0.001) but not in LPS-treated bEnd.3 cells in mono-culture (0.04 ± 0.02 vs 0.07 ± 0.03, P>0.05) (S3A and S3B Fig). In LPS-treated APP mice, fibrinogen immunoreactivity was found only in the blood vessels but not in the brain parenchyma. The ratio of fibrinogen: CD31 immunostaining in the blood vessels was 0.19 ± 0.03% in LPS-treated APP mice and that in PBS-treated APP mice was 0.02 ± 0.01%, P < 0.001 (Fig 6A and 6B). Thus, increased fibrinogen deposition was limited to the brain endothelial cells under the condition of the dose and administration route of LPS, suggesting that disruption of BBB did not occur, which is consistent with another recent study [61]. However, because fibrinogen attached to endothelial cells connects leukocytes to endothelial cells [62] and because inflammatory cytokines are upregulated in bEnd.3 cells co-cultured with LPS-activated Raw 264.7 cells, expression levels of cytokines that are upregulated in bEnd.3 cells co-cultured with LPS-activated Raw 264.7 cells (Figs 2 and 3) were determined by RT-PCR analysis in the hippocampi of LPS- or PBS-treated APP mice. The levels of CCL2 (P = 0.0096), CCL5 (P < 0.001), CSF1 (P = 0.006), CXCL1 (P < 0.001), CXCL10 (P = 0.014), ICAM1 (P = 0.002), IL-1α (P = 0.003), IL-1β (P = 0.002), and IRF7 (P < 0.001) in LPS-treated APP mice were significantly higher than those in PBS-treated APP mice (Fig 6C), as in the case of the co-culture model. Although the level of claudin-5 in bEnd.3 cells co-cultured with LPS-treated Raw 264.7 cells decreased, such decreases are not observed in LPS-treated APP mice compared with their controls (S4A and S4B Fig). These results indicate that LPS-induced systemic inflammation mediates the adherence of fibrinogen to the brain blood vessels and elicits robust expression of inflammatory cytokines and chemokines in brain endothelial cells, which may cause brain inflammation.

## Discussion

Peripheral administration of LPS has been widely used to investigate the effect of systemic inflammation on neuroinfammation in rodents [32, 58, 63]. Peripheral LPS could cross the BBB, activate the cells in the CNS and/or indirectly induce CNS effects by many other means, such as stimulating vagal or other afferent nerves, inducing secretion of cytokines from the immune cells or from the cells constituting the BBB (reviewed in [64]). Banks *et al*. [65] conducted intraperitoneal injection of different doses of LPS (0.03, 0.3, or 3 mg/kg) and found the highest dose of LPS (3mg/kg) significantly increased the BBB permeability but the lower doses had no effect. Goncalves *et al*. [66] found that the systemic administration of LPS (3, 5 and 7 mg/kg, with intervals of 48h between injections) induced a transient BBB interruption. Endothelial cells, one of the components of the BBB, are first responders to peripheral inflammatory molecules including LPS. Therefore, LPS-treated endothelial cells (1 μg/ml) have been intensively studied to investigate the effects of peripheral inflammation on the BBB structure and function [26–28]. However, these dosages of LPS injection in animal models, which can disrupt the BBB integrity are far beyond the clinical concentrations naturally found in mice and humans with chronic or acute inflammatory conditions [67–69]. In this study, intraperitoneal administration of low dose LPS (0.5 mg/kg) was used to mimic a low-grade inflammatory condition. Moreover, to our knowledge, this is the first to use bEnd.3 cells co-cultured with LPS (100ng/ml)-stimulated Raw 264.7 cells as an in vitro model to study the consequence of LPS-stimulated macrophage-derived mediators on endothelial cells. Two different models (model 1 and model 2) were tested to optimize co-culture conditions in sham co-culture models as shown in S5A and S5C Fig. In the model 1, the levels of TNFα in the upper wells are lower than those in the bottom wells in the absence of FBS. In contrast to the model 1, the levels of TNFα in the upper wells are higher than those in the bottom wells in the presence of FBS in the model 2, indicating that the presence of FBS determines the distribution of TNFα between the upper and bottom wells. Similarly, in our co-culture model 2 in S5B and S5D Fig, the levels of TNFα in the upper wells are higher than those in the bottom wells, indicating that the distribution of TNFα to the upper well is not influenced by bEnd.3 cells in the upper wells, which do not form a barrier structure as shown in S3A Fig. Such poor barrier functions of endothelial cell lines including bEnd.3 cells have been previously reported [70]. Therefore, the model 2 in S5B and S5D Fig was employed as a co-culture system in this study, where the endothelial cells on the upper wells are readily exposed to cytokines produced from LPS-stimulated macrophages. We found that among cytokines analyzed, eight cytokines/chemokines were commonly upregulated in both in vivo mouse and in vitro co-culture models, including G-CSF, IL-1ra, IL-6, CXCL10, CXCL1, CCL2, TIMP-1, and TNFα. In contrast to the co-culture model, the cytokine profile of the bEnd.3 cell mono-culture model bore little or no similarity to that of the in vivo mouse model (S1 Fig). These results indicate that the co-culture of bEnd.3 cells with LPS-activated Raw 264.7 cells is a better model than the mono-culture of LPS-stimulated bEnd.3 cells to investigate the molecular mechanism/signaling pathways in brain endothelial cells.

Cytokines, a broad family of intercellular signaling proteins, play an important role in the regulation of inflammation and infection in the body [71]. Cytokine signaling at the BBB mediates the dynamic regulation of its function and influences brain homeostasis and disease [72]. The increase in serum IL-6 due to periodontal disease reduced claudin-5 levels in the brain blood vessels, resulting in increased BBB permeability [73]. Zhang *et al*. [74] reported that anti-IL-6 mAb infusion attenuated increases in BBB permeability by IL-6. TNFα has been shown to increase permeability in brain-like endothelial cells (BLECs) through the paracellular pathway by monitoring the passage rate of small hydrophilic marker lucifer yellow [75]. Additionally, many other cytokines were reported to influence the BBB function, including G-CSF [76], CXCL1 [77] and IL-1β [75, 78]. Although a reductionist approach with mono-culture of endothelial cells is useful to identify (a) specific pathway(s) activated by a single-cytokine in endothelial cells, many cytokines are secreted in systemic inflammation and infectious diseases as exemplified by cytokine storm induced by the severe acute respiratory syndrome coronavirus 2 (SARS-CoV-2) [79]. Therefore, an endothelial mono-culture system by LPS cannot mimic inflammatory mediators induced by systemic inflammation and infection as shown in Fig 2 and S1 Fig. We have established a co-culture system of endothelial and immune (macrophage) cells to investigate the signaling mechanisms activated in endothelial cells by macrophage-derived inflammatory mediators by LPS, which may modulate the BBB integrity. COVID-19-asssociated cytokine storm has been proposed to cause neurological disorders via BBB dysfunction and SARS CoV2 neuroinvasion [80]. The co-culture system may be useful to investigate the signaling mechanisms underlying the neurological disorders associated with COVID-19, also.

Our RNA-seq data demonstrated that cytokines secreted from LPS-treated Raw 264.7 cells activated cytokine signaling pathways in bEnd.3 cells. It is known that cytokines mediate innate and adaptive immune responses and that they signal via their cell surface receptors and activate JAK/STAT or MAPK pathways [81]. The RNA-seq and western blot analysis of bEnd.3 cells co-cultured with LPS-activated Raw 264.7 cells showed increased mRNA and protein levels of both STAT1 and STAT2. STAT activity is especially high in cells of the BBB, in which it causes abnormal BBB permeability [82]. These results suggest that STAT signaling may be a potential target to modulate the systemic inflammation-induced BBB dysfunction. JAK/STAT inhibitors have been widely studied [81] and several JAK inhibitors have been approved by FDA for the treatment of rheumatoid arthritis, myelofibrosis, and atopic dermatitis [83].

LPS-treated rodents have been widely used to investigate potential roles of neuroinflammation in the pathogenesis of neurological diseases including AD [58, 63, 84]. Animals’ responses to LPS widely vary with dose, LPS source, route and frequency of administration, species, strain, sex and age [58]. Catorce and Gevorkian [63] reviewed LPS-induced murine neuroinflammation models and suggested that peripherally induced inflammation by LPS may trigger neuroinflammation leading to Aβ and/or tau pathology, neurodegeneration and cognitive impairment. Considering increased BBB permeability by LPS [55, 65] and fibrinogen infiltration via a leaky BBB [85], we investigated the BBB permeability by immunofluorescence microscopy using antibodies specific to fibrinogen and CD31, a marker for endothelial cells. We found that fibrinogen fluorescence signals were increased and exclusively limited to the areas positive for CD31 fluorescence signals in LPS-treated mice (Fig 6), indicating that LPS treatment did not induce infiltration of fibrinogen into the brain parenchyma. Fibrinogen, an abundant protein synthesized in the liver, is present in blood and increases in infections [86, 87]. Because fibrinogen plays multifaceted roles in tissue injury and inflammation [88], elevated levels of fibrinogen may cause vascular dysfunctions, including increases in blood viscosity and vascular reactivity, and decreases in endothelial integrity [89]. Although we did not find infiltration of fibrinogen in the brain parenchyma, intraperitoneal administration of 0.5 mg/kg LPS decreased soluble Aβ 40 and 42 levels in the brain and increased those in the CSF, indicating that LPS treatment increased soluble Aβ efflux from brain parenchyma to CSF. It is also possible that the decreased Aβ levels are due to increased expression levels of angiotensin I converting enzyme (ACE), which is observed in the brain in LPS-treated APP mice (S6 Fig). ACE has been reported to degrade Aβ in vitro [90, 91] and ACE-overexpression in AD mice reduces Aβ load [92]. Many cytokines that increased in the hippocampi of LPS-treated APP mice are also increased in bEnd.3 cells co-cultured with LPS-activated Raw 264.7 cells. However, the degree of the contribution from brain endothelial cells to the increased expression levels of cytokines and chemokines in LPS-treated APP mice is uncertain because bulk RNAs from a number of different cell types in the hippocampi were used. We found that LPS-induced acute systemic inflammation enhances the adhesion of fibrinogen to brain endothelial cells in vivo, which is in line with the observation by the in vitro co-culture model where LPS-activated macrophages induced attachment of fibrinogen to endothelial cells. Because fibrinogen attached to brain endothelial cells can capture leukocytes and facilitate their infiltration into the brain [62], repeated systemic inflammation or chronic systemic inflammation may disrupt the integrity of the BBB by enhancing neuroinflammation. Together, LPS-induced cytokines and fibrinogen may stimulate endothelial cells to further express chemokines/cytokines via cytokine signaling pathways. Fibrinogen binds to ICAM1 that was increased in endothelial cells in the co-culture model as well as in vivo model, which may enhance endothelial inflammation by bridging leukocyte adherence to endothelial cells [93], leading to the BBB dysfunction.

It is worth noting that endothelial cells alone are not sufficient to establish a barrier structure and additional cell types of the BBB such as astrocytes and pericytes are required to achieve the complete barrier [70, 94, 95]. Therefore, our co-culture model with bEnd.3 cells has limitations in studying the endothelial cell permeability and blood-brain barrier function.

## Conclusions and limitations

The in vitro LPS-treated Raw 264.7/macrophages and bEnd.3 cells co-culture model far better mirrors the interaction of inflammatory stimuli derived from LPS-activated macrophages and endothelial cells lining the interior surface of the blood vessels in vivo than LPS-treated endothelial cells mono-culture model. Although peripheral inflammatory conditions may exert detrimental effects on the endothelium through signaling complexes in vivo, GO analysis based on DEGs reveals that JAK/STAT signaling pathways play dominant roles in the endothelial cells of the co-culture model of LPS-induced inflammation. In vivo experimental findings suggest that acute peripheral inflammation by LPS increases the efflux of Aβ from the brain to the CSF. In the future, we will investigate whether STAT1/2 inhibitors alleviate the effects of peripheral inflammation on the BBB and central nervous system. However, LPS-induced-Raw 264.7/macrophages and bEnd.3 cells co-culture models are limited to study the effect of systemic inflammation on brain endothelial cells and, partially but not fully on the BBB that are composed of several different cell types.

## Supporting information

S1 Fig LPS induces inflammatory cytokines secretion in vivo. The levels of TNFα and IL-6 in plasma at 2h and/or 24h after LPS injection (A) (n = 6 for each group). Analysis of chemokines/cytokines in plasma (B, C, & D) (n = 3 for each group) by proteome profiler mouse cytokine array. (D) Semi quantitative analysis of chemokines/cytokines. The identification numbers labeled right above or below or on the right of the signal dots in S1 Fig. B and C correspond to the numbers assigned to cytokines and chemokines in S1 Fig. D (#P < 0.01, and ##P < 0.001).
(TIF)

S2 Fig LPS does not alter the levels of STAT and Claudin-5 in bEnd.3 cells mono-culture. The bEnd.3 cell lysates were analyzed by western blotting using Stat1, Stat2, and Claudin-5 antibodies (A, C, and E) and bar graph represents the results of densitometric analysis (B, D, and F). Representative blots from three independent experiments are shown.
(TIF)

S3 Fig LPS increases fibrinogen binding to the bEnd.3 cells co-cultured with LPS-treated Raw 264.7 cells (A), Immunofluorescent staining of endothelial marker, CD31 (red) and fibrinogen (green). Scale bar: 20μm. (B), The ratios of fibrinogen positive cells to total cells. (## P < 0.001).
(TIF)

S4 Fig LPS does not alter the levels of brain claudin-5 in APP mice. The left neocortex lysates were analyzed by western blotting using Claudin-5 antibody (A) and bar graph represents the levels of normalized Claudin-5 by denstitometric analysis (B) (n = 6 male mice/group).
(TIF)

S5 Fig FBS-dependent distribution of cytokines produced by LPS-activated Raw 264.7 cells. (A), Sham co-culture model 1 and model 2 contained Raw 264.7 cells at the bottom well and, on the day 3, LPS (100ng/ml) was added to the bottom of well in the absence or presence of FBS, respectively. (B), Co-culture model 1 and model 2 had bEnd.3 cells at the upper well and Raw 264.7 cells at the bottom well and, on the day 3, LPS (100ng/ml) was added to the bottom of well in the absence or presence of FBS, respectively. (C), In sham co-culture, the levels of TNFα in the upper wells are significantly lower than those in the bottom wells in model 1 (P<0.05, the ratio of the upper to bottom: 0.85). The levels of TNFα in the upper wells are significantly higher than those in the bottom wells in model 2 (P<0.05, the ratio of the upper to bottom: 1.27). (D), In co-culture, the levels of TNFα in the upper wells are significantly lower in model 1 (P<0.05, the ratio of the upper to bottom: 0.61) but higher in model 2 (P<0.05, the ratio of the upper to bottom: 1.37) than those in the bottom wells.
(TIF)

S6 Fig LPS-treated APP mice show increased ACE in the brain. Quantitative real-time PCR was used to determine expression levels of the genes in the hippocampus, which are known to modulate Aβ levels and include β-Secretase 1 (BACE1), presenilin 1 (PS1), insulin degrading enzyme (IDE), angiotensin I converting enzyme (ACE), and neprilysin (NEP). The bar graph represents the relative expression levels of the genes. (n = 6 male mice/group, #P < 0.01).
(TIF)

S1 Data Supporting methods and materials. (DOCX)

S1 Table (XLSX)

S2 Table (XLSX)

S3 Table (XLSX)

S4 Table (XLSX)

S1 Raw images (PDF)
