# Supplementary figures and images for: A novel co-culture model for investigation of the effects of LPS-induced macrophage-derived cytokines on brain endothelial cells
Source: PLoS One. 2023 Jul 13;18(7):e0288497. doi: 10.1371/journal.pone.0288497 (PMC10343049; doi:10.1371/journal.pone.0288497)

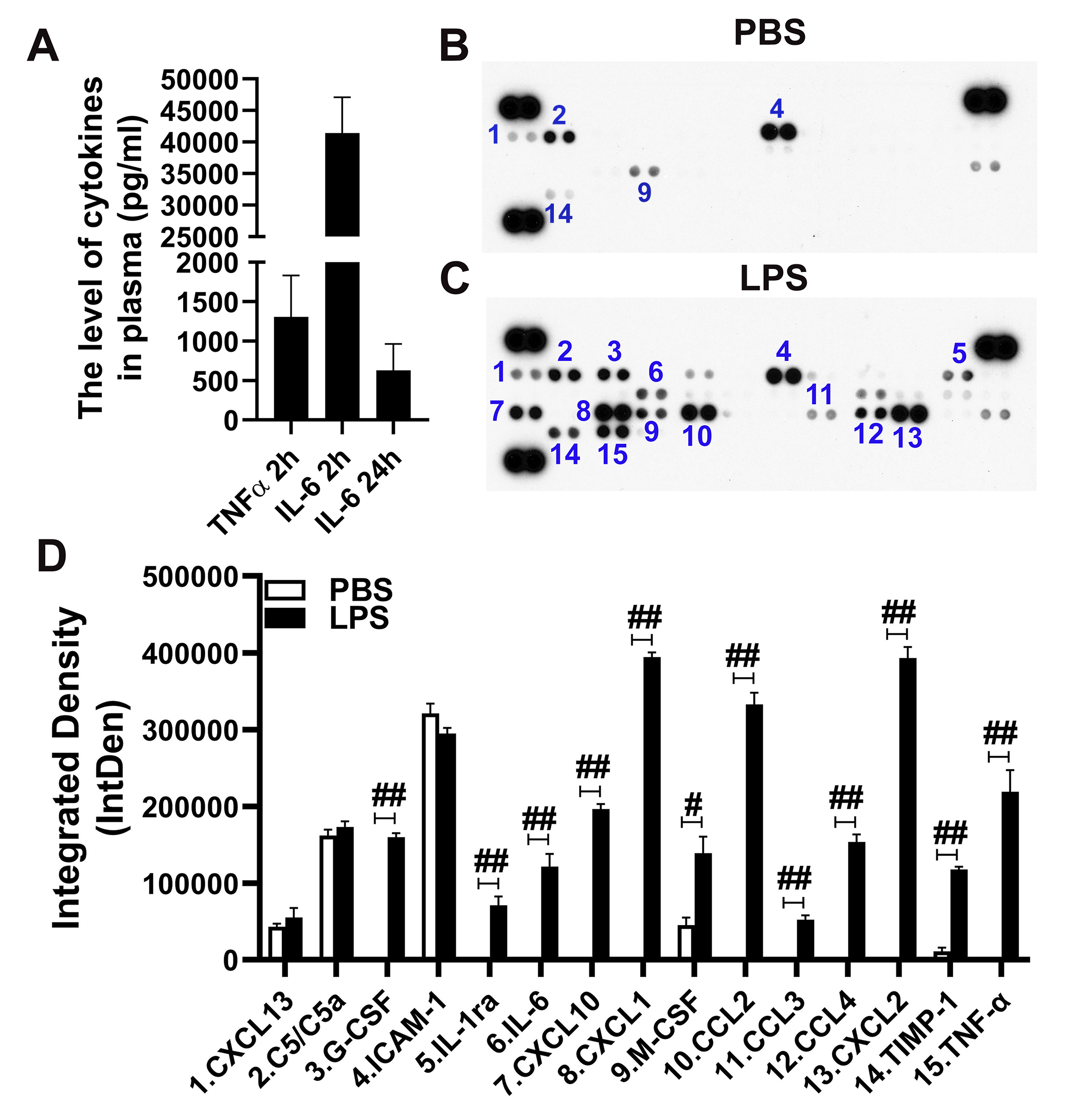

Supplement: S1 Fig — The levels of TNFα and IL-6 in plasma at 2h and/or 24h after LPS injection (A) (n = 6 for each group). Analysis of chemokines/cytokines in plasma (B, C, & D) (n = 3 for each group) by proteome profiler mouse cytokine array. (D) Semi quantitative analysis of chemokines/cytokines. The identification numbers labeled right above or below or on the right of the signal dots in S1 Fig. B and C correspond to the numbers assigned to cytokines and chemokines in S1 Fig. D (#P < 0.01, and ##P < 0.001). (TIF) [file pone.0288497.s001.tif]

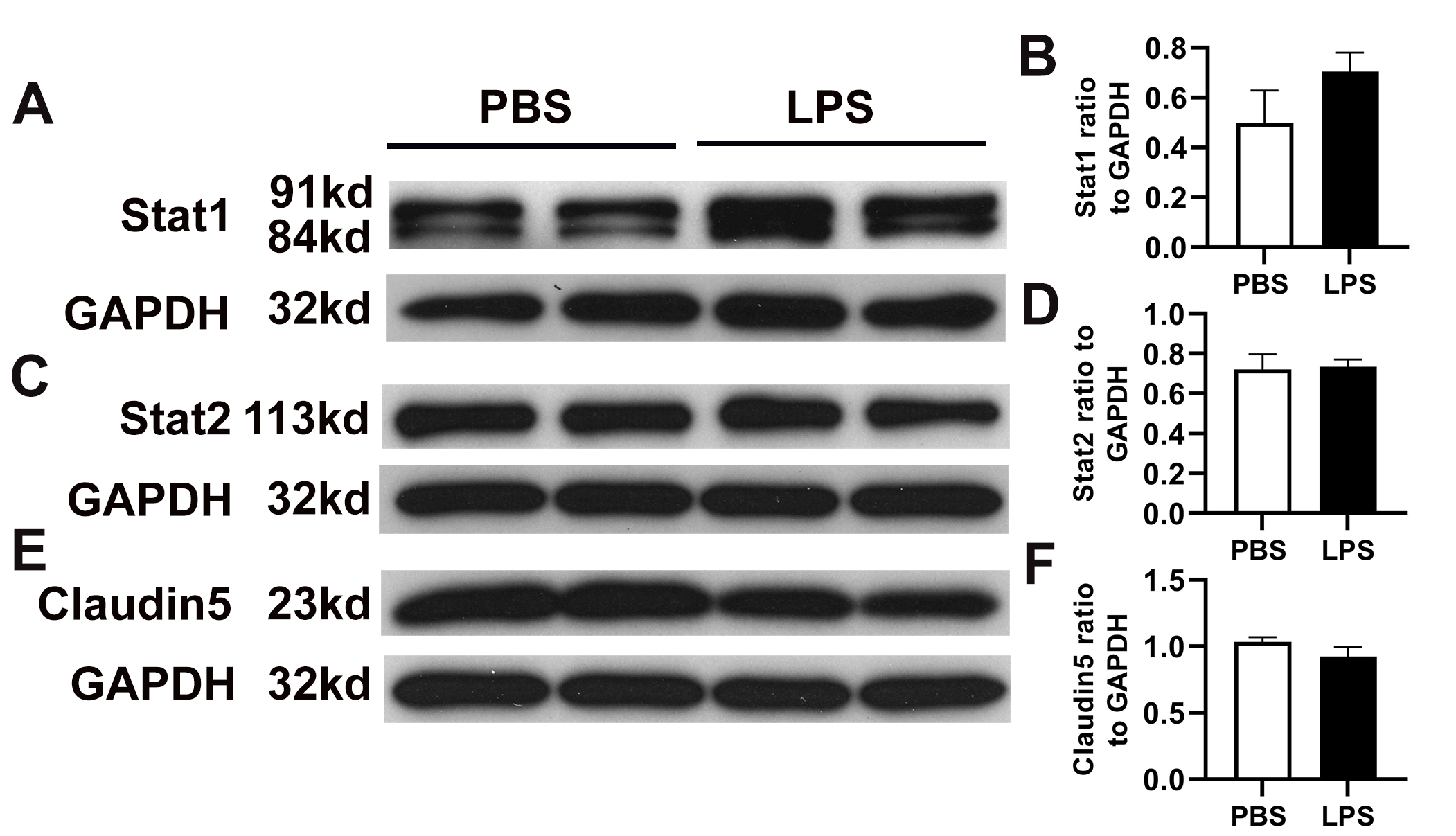

Supplement: S2 Fig — The bEnd.3 cell lysates were analyzed by western blotting using Stat1, Stat2, and Claudin-5 antibodies (A, C, and E) and bar graph represents the results of densitometric analysis (B, D, and F). Representative blots from three independent experiments are shown. (TIF) [file pone.0288497.s002.tif]

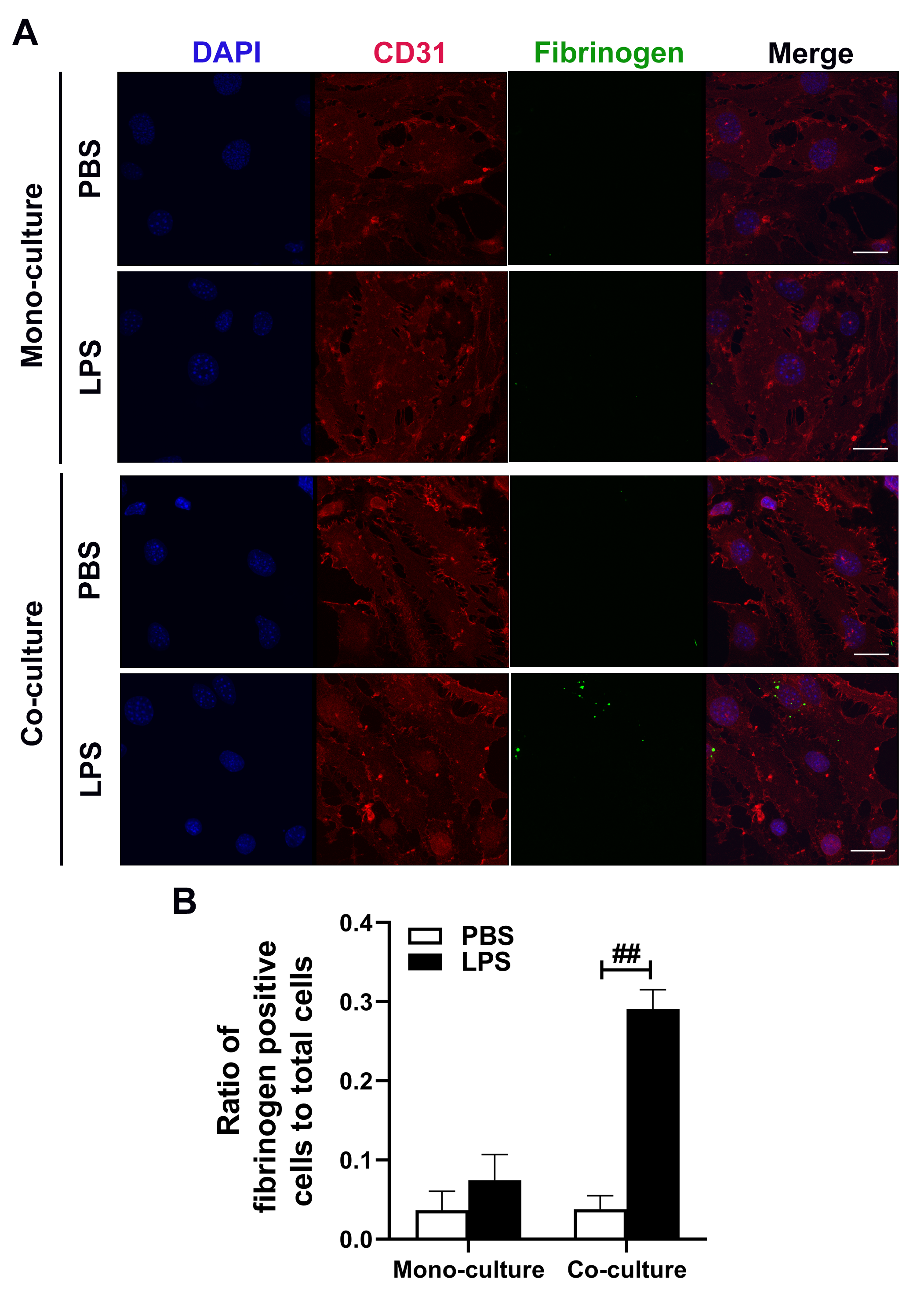

Supplement: S3 Fig — LPS increases fibrinogen binding to the bEnd.3 cells co-cultured with LPS-treated Raw 264.7 cells (A), Immunofluorescent staining of endothelial marker, CD31 (red) and fibrinogen (green). Scale bar: 20μm. (B), The ratios of fibrinogen positive cells to total cells. (## P < 0.001). (TIF) [file pone.0288497.s003.tif]

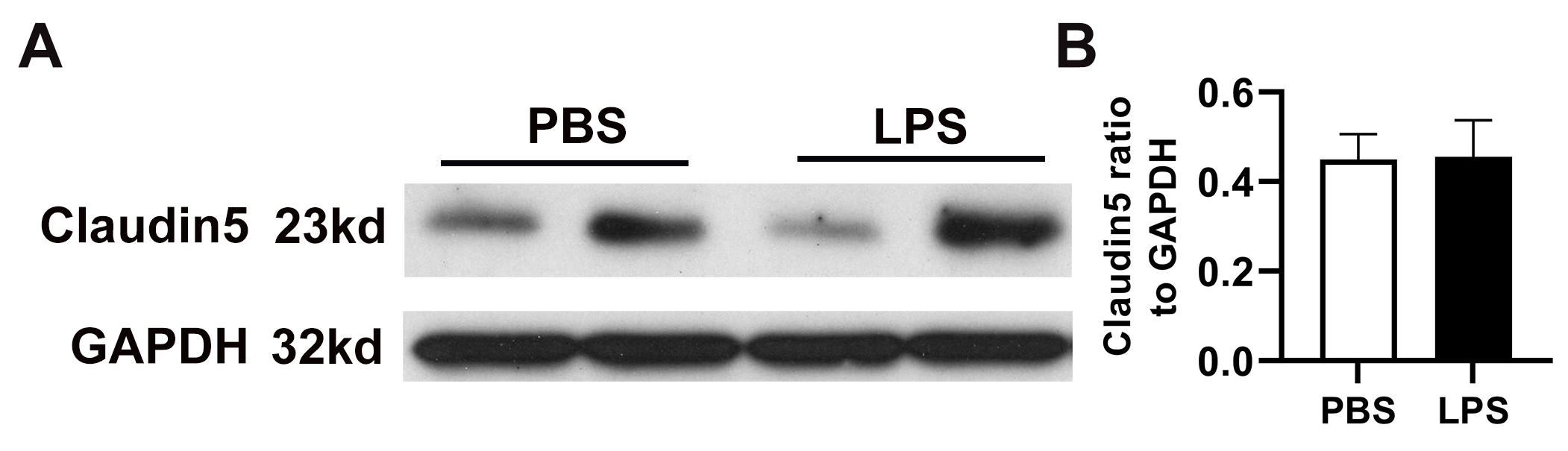

Supplement: S4 Fig — The left neocortex lysates were analyzed by western blotting using Claudin-5 antibody (A) and bar graph represents the levels of normalized Claudin-5 by denstitometric analysis (B) (n = 6 male mice/group). (TIF) [file pone.0288497.s004.tif]

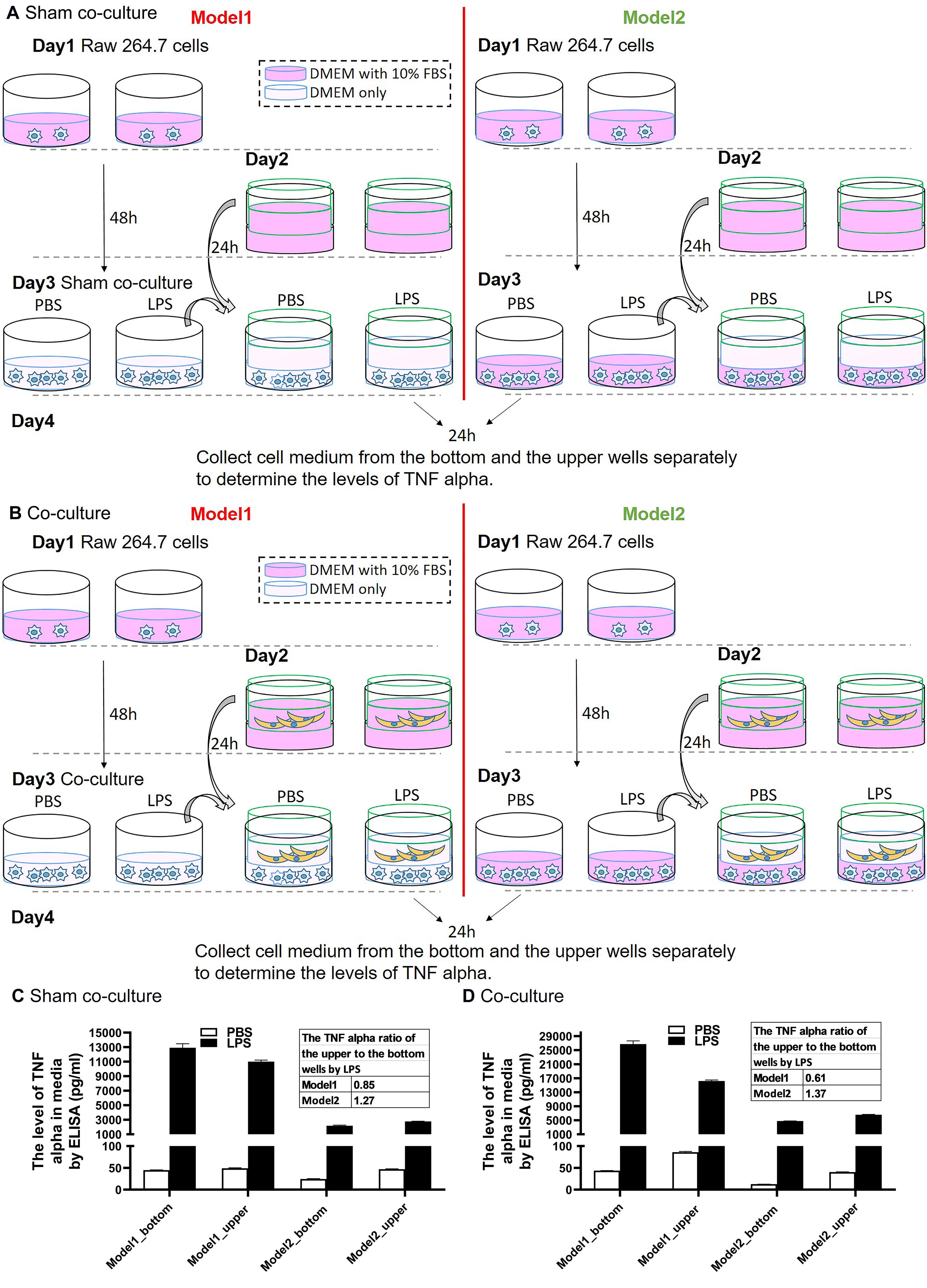

Supplement: S5 Fig — (A), Sham co-culture model 1 and model 2 contained Raw 264.7 cells at the bottom well and, on the day 3, LPS (100ng/ml) was added to the bottom of well in the absence or presence of FBS, respectively. (B), Co-culture model 1 and model 2 had bEnd.3 cells at the upper well and Raw 264.7 cells at the bottom well and, on the day 3, LPS (100ng/ml) was added to the bottom of well in the absence or presence of FBS, respectively. (C), In sham co-culture, the levels of TNFα in the upper wells are significantly lower than those in the bottom wells in model 1 (P<0.05, the ratio of the upper to bottom: 0.85). The levels of TNFα in the upper wells are significantly higher than those in the bottom wells in model 2 (P<0.05, the ratio of the upper to bottom: 1.27). (D), In co-culture, the levels of TNFα in the upper wells are significantly lower in model 1 (P<0.05, the ratio of the upper to bottom: 0.61) but higher in model 2 (P<0.05, the ratio of the upper to bottom: 1.37) than those in the bottom wells. (TIF) [file pone.0288497.s005.tif]

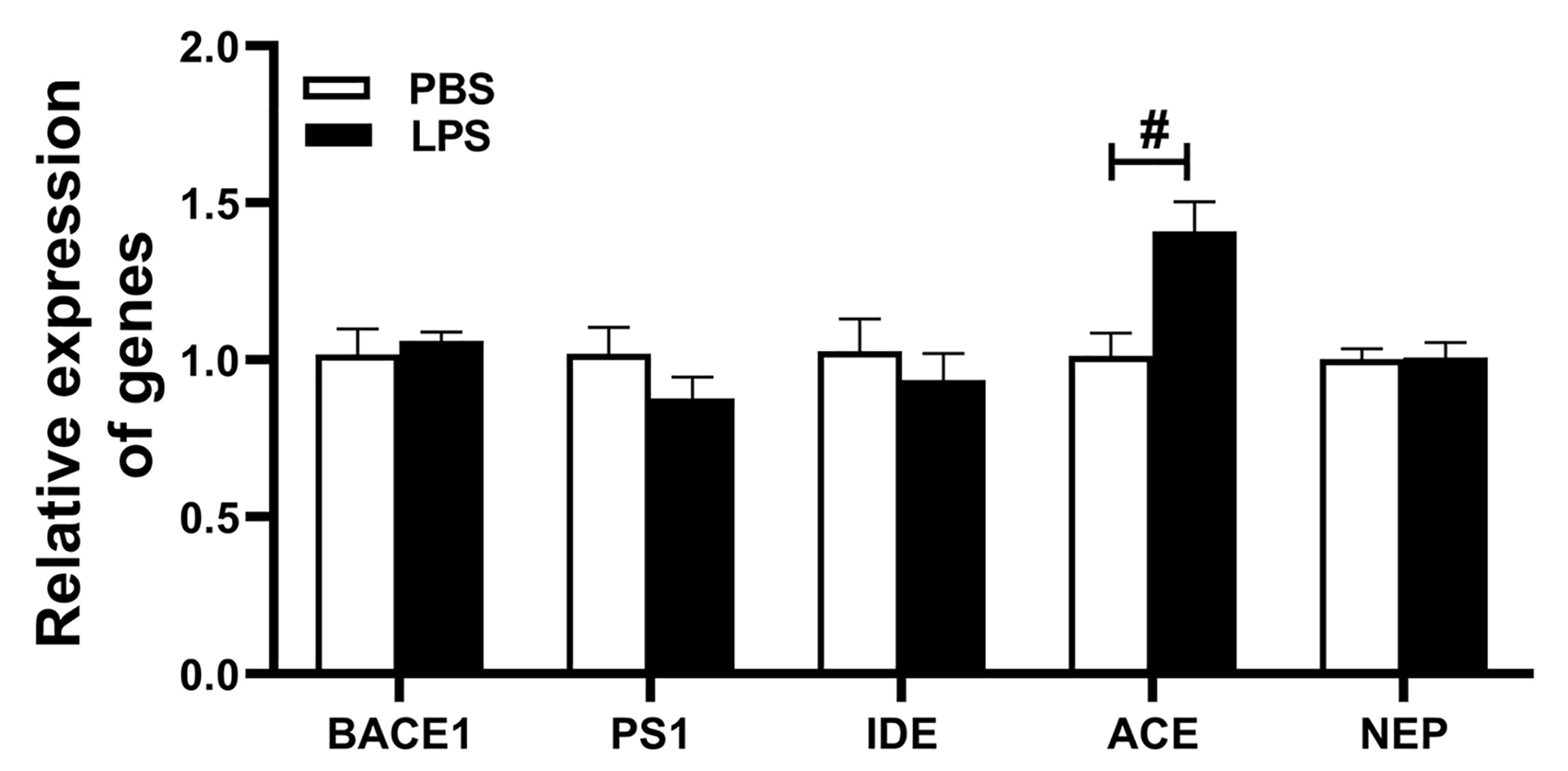

Supplement: S6 Fig — Quantitative real-time PCR was used to determine expression levels of the genes in the hippocampus, which are known to modulate Aβ levels and include β-Secretase 1 (BACE1), presenilin 1 (PS1), insulin degrading enzyme (IDE), angiotensin I converting enzyme (ACE), and neprilysin (NEP). The bar graph represents the relative expression levels of the genes. (n = 6 male mice/group, #P < 0.01). (TIF) [file pone.0288497.s006.tif]
