# Supplementary material for: A novel co-culture model for investigation of the effects of LPS-induced macrophage-derived cytokines on brain endothelial cells
Source: PLoS One. 2023 Jul 13;18(7):e0288497. doi: 10.1371/journal.pone.0288497 (PMC10343049; doi:10.1371/journal.pone.0288497)
Supplement: S1 Data — (DOCX) [file pone.0288497.s007.docx]

**Supporting methods and materials**

**Quantification of cytokines expression in plasma by cytokine array and ELISA**

Approximately 100 µl of blood/mouse (14-month-old APP mice) was drawn from the tail vein before and 2h and 24h after LPS or PBS injection by use of microhematocrit heparin tubes (Thermo Fisher Scientific, Waltham, MA) and centrifuged for 20 min at 2,000 x g for plasma isolation. Plasma samples were used to determine the levels of TNFα and IL-6 by ELISA kits (eBioscience, Inc. San Diego, CA) according to the manufacturer’s protocol. Plasma samples from 6-month-old APP mice (50ul/mouse) were used for cytokine profiling analysis, using a proteome profiler mouse cytokine array kit, panel A (R&D Systems, Inc. Minneapolis, MN).

**Fibrinogen binding assay in cell culture model**

Raw 264.7 cells and bEnd.3 cells were seeded as outlined in Fig 1. On day 3, RAW 264.7 cells were treated with PBS or 100ng/ml LPS in 10% FBS DMEM. Meanwhile, the media in the transwell inserts with bEnd.3 cells were replaced with fresh DMEM containing 15ug/ml of Alexa Fluor 488 conjugated fibrinogen (Cat# F13191, Thermo Fisher Scientific, Waltham, MA) and bEnd.3 cells in the transwell inserts were co-cultured with Raw 264.7 cells treated with PBS or LPS. As for mono-culture system, the conditions were same except no Raw 264.7. Twenty-four hours after LPS treatment, bEnd.3 cells on the transwell inserts were rinsed twice with PBS, and then fixed with 4% PFA for 15min at room temperature followed by washing with PBS, 3 times, 10min each at room temperature. The membranes with bEnd.3 cells were detached from the transwell using a scalpel and incubated in 0.1 M TBS with 0.5% triton-X-100 (TBS-T) and 1% BSA for 1h at room temperature and then incubated with CD31 primary antibody (Cat# 557355, 1: 500 dilution, BD Pharmingen™, Franklin Lakes, NJ) overnight at 4 ̊C. For the negative controls, membranes were processed without the primary antibody. After rinsing, the membrane were incubated with chicken-anti-rat antibody (Thermo Fisher Scientific, Waltham, MA) for 2 h at room temperature. Cell nuclei were counterstained with DAPI. The membranes were mounted in ShandonTM Immu-MountTM (Thermo Fisher Scientific, Waltham, MA). Histomorphometry was performed by determining the ratios of fibrinogen positive cells to the total number of cells in randomly captured 15 areas using Olympus FV3000 confocal microscope (Olympus American Inc, Waltham, MA). Around 100 cells were counted per samples.
