# Supplementary material for: A novel co-culture model for investigation of the effects of LPS-induced macrophage-derived cytokines on brain endothelial cells
Source: PLoS One. 2023 Jul 13;18(7):e0288497. doi: 10.1371/journal.pone.0288497 (PMC10343049; doi:10.1371/journal.pone.0288497)
Supplement: S1 Raw images — (PDF) [file pone.0288497.s012.pdf]

Fig 2A, B, D, and E

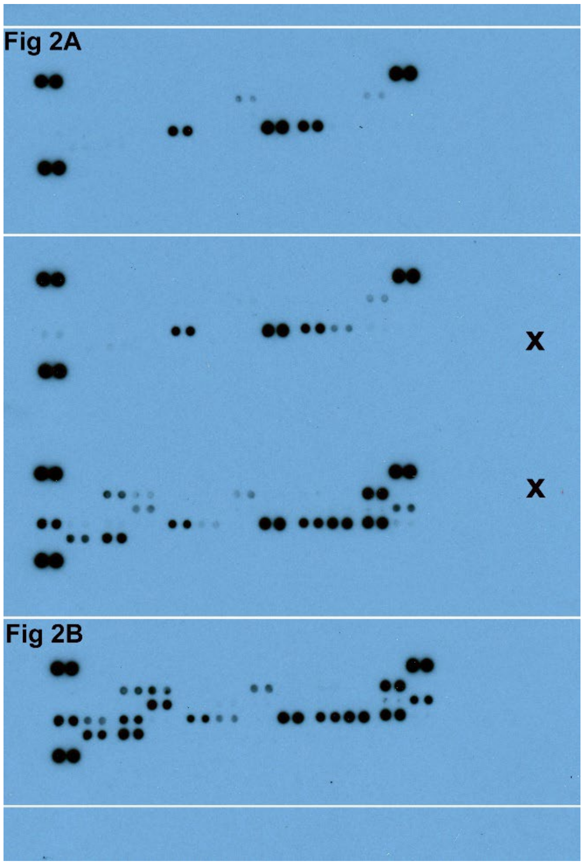

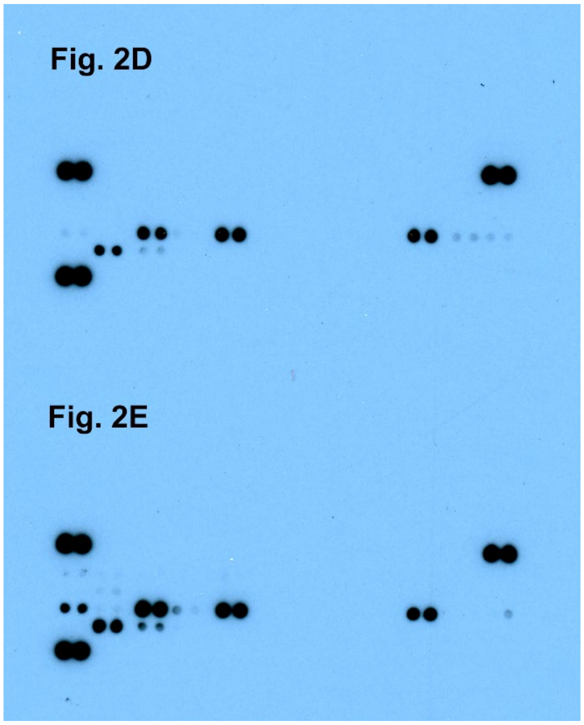

Fig.4A, C, E, and G

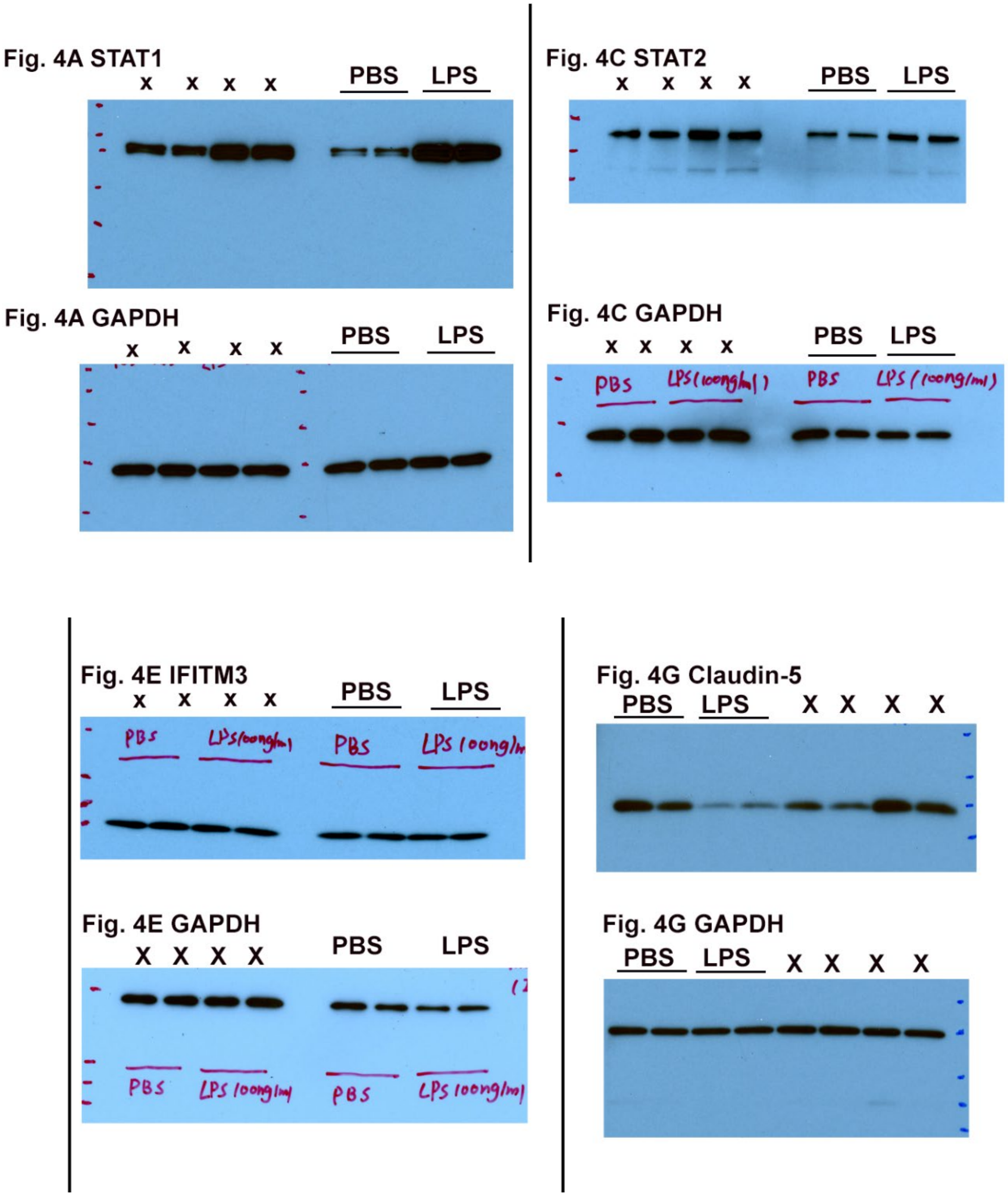

S1 Fig. B and C

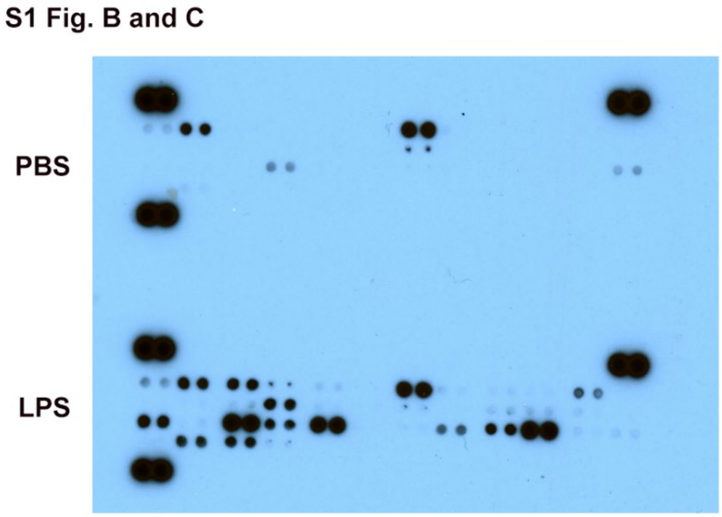

S2 Fig. A, C and E

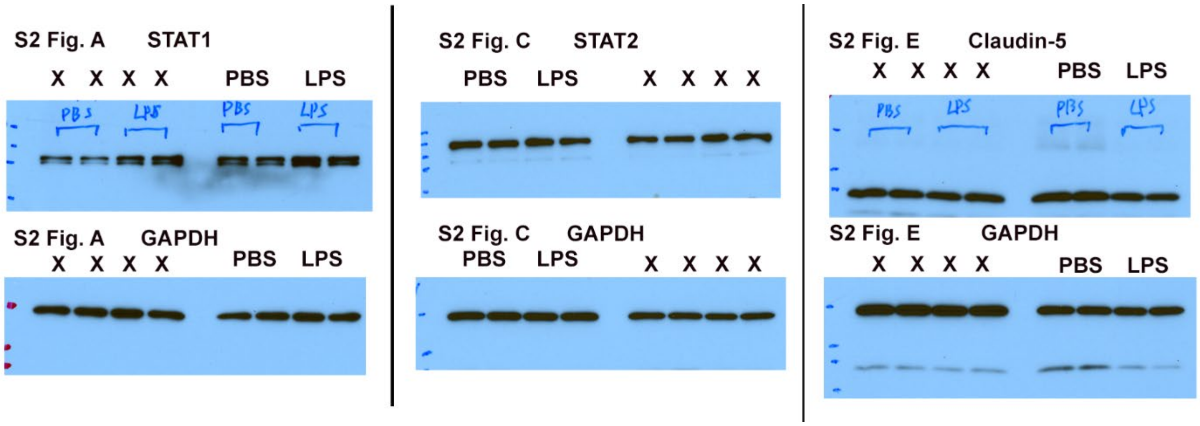

S4 Fig. A

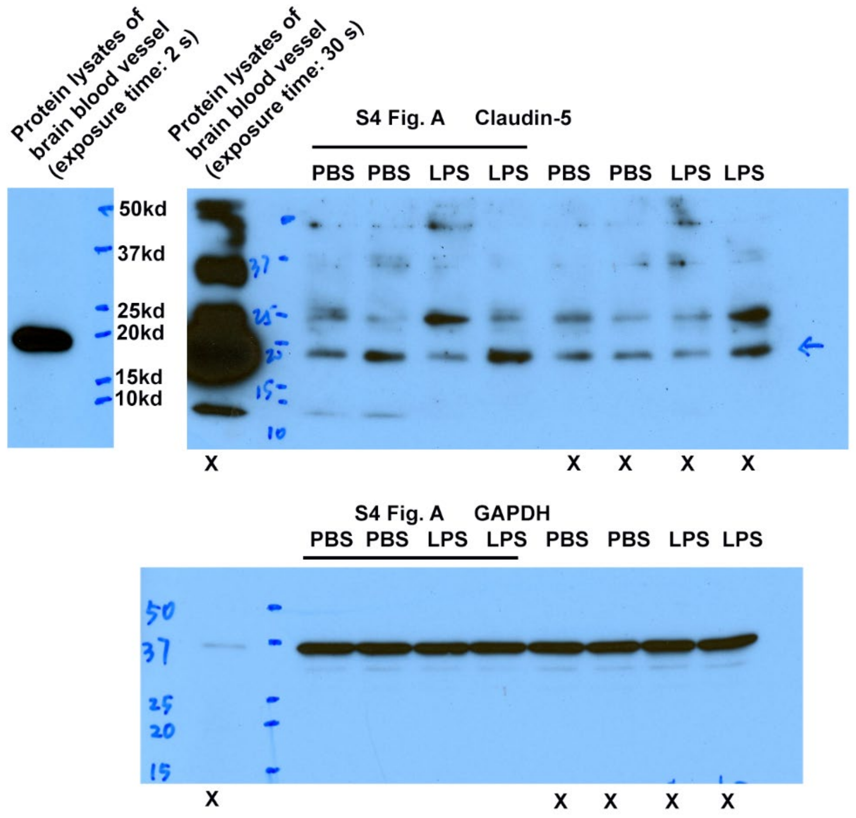

Protein lysates of brain blood vessel (exposure time: 2 s)
Protein lysates of brain blood vessel (exposure time: 30 s)
S4 Fig. A Claudin-5
PBS PBS LPS LPS PBS PBS LPS LPS
50kd
37kd
25kd
20kd
15kd
10kd
X
X X X X
S4 Fig. A GAPDH
PBS PBS LPS LPS PBS PBS LPS LPS
50
37
25
20
15
X
X X X X
